# Supplementary material for: Structural insights into anion selectivity and activation mechanism of LRRC8 volume-regulated anion channels
Source: Cell Rep. 2023 Aug 6;42(8):112926. doi: 10.1016/j.celrep.2023.112926 (PMC10480491; doi:10.1016/j.celrep.2023.112926)
Supplement: Document S1. Figures S1–S9 and Tables S1–S3 [file mmc1.pdf]

Cell Reports, Volume *42*

Supplemental information

Structural insights into anion selectivity and activation mechanism of LRRC8 volume-regulated anion channels

Heng Liu, Maya M. Polovitskaya, Linlin Yang, Meiling Li, Hongyue Li, Zhen Han, Jianguo Wu, Qiansen Zhang, Thomas J. Jentsch, and Jun Liao

Figure S1

```
                    NT                  TM1                      E1β
LRRC8A_Homo     MIPVTELRYFADTQPAYRILKPWWDVFTDYISIVMLMIAVFGGTLQVTQDKMI-CLPCKW  59
LRRC8A_Mus      MIPVTELRYFADTQPAYRILKPWWDVFTDYISIVMLMIAVFGGTLQVTQDKMI-CLPCKW  59
LRRC8A_Gallus   MIPVTELRYFADTQPAYRILKPWWDVFTDYISIVMLMIAVFGGTLQVTQDKMI-CLPCKW  59
LRRC8A_Xenopus  MIPVTELRYFADTQPAYRILKPWWDVFTDYISIVMLMIAVFGGTLQVTQDKMI-CLPCKW  59
LRRC8D_Homo     MFTLAEVASLNDIQPTYRILKPWWDVFMDYLAVVMLMVAIFAGTMQLTKDQVV-CLPVLP  59
LRRC8C_Homo     MIPVTEFRQFSEQQPAFRVLKPWWDVFTDYLSVAMLMIGVFGCTLQVMQDKII-CLPKRV  59
LRRC8E_Homo     MIPVAEFKQFTEQQPAFKVLKPWWDVLAEYLTVAMLMIGVFGCTLQVTQDKII-CLPNHE  59
LRRC8B_Homo     MITLTELKCLADAQSSYHILKPWWDVFWYYITLIMLLVAVLAGALQLTQSRVLCCLPCKV  60

LRRC8A_Homo     VTKDSCNDSFRGWAA-PG---------------------------PEPTYP------N--  83
LRRC8A_Mus      VTKDSCNDSFRGWAA-SN---------------------------PEPTYP------N--  83
LRRC8A_Gallus   ITKDSCNDTVRGWTA-VT---------------------------PERIYY------N--  83
LRRC8A_Xenopus  VTHDSCNDSYRAWNV-PE---------------------------TD-LYT------N--  82
LRRC8D_Homo     SPVNSKAHTPPGNAEVTTNIPKMEAATNQDQDGRTTNDISFGTSAVTPDIPLRATYPRTD 119
LRRC8C_Homo     QPAQNHSSLSNVSQAVAS---------------------------TTPLPPP-----K--  85
LRRC8E_Homo     LQENL-------SEA-PC---------------------------QQLLPRG-----I--  77
LRRC8B_Homo     EFDNHCAVPWDILKA-SM---------------------------NTSSNP---------  83
                                               E1H          TM2
LRRC8A_Homo     ----STILPTPDTGPTGIKYDLDRHQYNYVDAVCYENRLHWFAKYFPYLVLLHTLIFLAC 139
LRRC8A_Mus      ----STVLPTPDTGPTGIKYDLDRHQYNYVDAVCYENRLHWFAKYFPYLVLLHTLIFLAC 139
LRRC8A_Gallus   ----SSLVPSPDTGPTGIQYDLDRHQYNYVDAVCYENRLHWFAKYFPYLVLLHTLIFLAC 139
LRRC8A_Xenopus  ----STLSPPLAPGPTGIKYDLDRHQYNYVDAVCYENRLHWFAKYFPYLVLLHTLIFLAC 138
LRRC8D_Homo     FALPNQEAKKEKKDPTGRKTNLDFQQYVFINQMCYHLALPWYSKYFPYLALIHTIILMVS 179
LRRC8C_Homo     ----PSPANPITVEMKGLKTDLDLQQYSFINQMCYERALHWYAKYFPYLVLIHTLVFMLC 141
LRRC8E_Homo     ----P-EQIGALQEVKGLKNNLDLQQYSFINQLCYETALHWYAKYFPYLVVIHTLIFMVC 132
LRRC8B_Homo     --------GTPLPLPLRIQNDLHRQQYSYIDAVCYEKQLHWFAKFFPYLVLLHTLIFAAC 135
                 TM2      IL1H1
LRRC8A_Homo     SNFWFKFPRTSSKLEHFVSILLKCFDSPWTTRALSETVVEESDPKPAFSKMN-GSMDKKS 198
LRRC8A_Mus      SNFWFKFPRTSSKLEHFVSILLKCFDSPWTTRALSETVVEESDPKPAFSKMN-GSMDKKS 198
LRRC8A_Gallus   SNFWFKFPRTSSKLEHFVSILLKCFDSPWTTRALSETVVEESDTKPAFGKMN-GSMDKKS 198
LRRC8A_Xenopus  SNFWFKFPRTSSKLEHFVSILLKCFDSPWTTRALSETVVEESDPKPTGGKMN-GSVDKKS 197
LRRC8D_Homo     SNFWFKYPKTCSKVEHFVSILGKCFESPWTTKALSETACEDSEENKQRITGAQTLP-KHV 238
LRRC8C_Homo     SNFWFKFPGSSSKIEHFISILGKCFDSPWTTRALSEVSGEDSEEKDNRKNNMNRSNTI-Q 200
LRRC8E_Homo     TSFWFKFPGTSSKIEHFISILGKCFDSPWTTRALSEVSGENQKGPAATERAAATIVAMAG 192
LRRC8B_Homo     SNFWLHYPSTSSRLEHFVAILHKCFDSPWTTRALSETVAEQSVRPLKLSKSK-IL--LSS 192
                                                                  IL1H2
LRRC8A_Homo     STVSEDV--EAT--VPMLQRTKSRIEQGIVDRSETGVLDKKEGEQAKALFEKVKKFRTHV 254
LRRC8A_Mus      STVSEDV--EAT--VPMLQRTKSRIEQGIVDRSETGVLDKKEGEQAKALFEKVKKFRTHV 254
LRRC8A_Gallus   STVSEDV--EAT--VPMLQRTKSRIEQGIVDRSETGVLDKKEGEQAKALFEKVKKFRTHV 254
LRRC8A_Xenopus  STASEDV--EAT--VPMLQRSKSRVEQGIVDRSETGVLDKKEGEQAKALFEKVKKFRTHV 253
LRRC8D_Homo     STSSDEGSPSASTPMINKTGFKFSAEKPVIEVPSMTILDKKDGEQAKALFEKVRKFRAHV 298
LRRC8C_Homo     SGP---E--GS---LVNSQSLKSIPEKFVVDKSTAGALDKKEGEQAKALFEKVKKFRLHV 252
LRRC8E_Homo     TGP---G--KAG--EGEKEKVLAEPEKVVTEPPVVTLLDKKEGEQAKALFEKVKKFRMHV 245
LRRC8B_Homo     SGCSADI--DSG--KQSLPYPQPGLESAGIESPTSSVLDKKEGEQAKAIFEKVKRFRMHV 248
                                   TM3                     E2β1      E2β2
LRRC8A_Homo     EEGDIVYRLYMRQTIIKVIKFILIICYTVYYVHNIKFDVDCTVDIESLTGYRTYRCAHPL 314
LRRC8A_Mus      EEGDIVYRLYMRQTIIKVIKFVLIICYTVYYVHNIKFDVDCTVDIESLTGYRTYRCAHPL 314
LRRC8A_Gallus   EEGDIVYRLYMRQTIIKVIKFILIICYTVYYVNNITFDVDCKVDIESLTGYRMYRCAHPL 314
LRRC8A_Xenopus  EEGDIVYRLYMRQTIIKVIKFIIILCYTVYYVSSIKFDVDCKVDIESLTGYRMYRCAHPL 313
LRRC8D_Homo     EDSDLIYKLYVVQTVIKTAKFIFILCYTANFVNAISFEHVCKPKVEHLIGYEVFECTHNM 358
LRRC8C_Homo     EEGDILYAMYVRQTVLKVIKFLIIIAYNSALVSKVQFTVDCNVDIQDMTGYKNFSCNHTM 312
LRRC8E_Homo     EEGDILYTMYIRQTVLKVCKFLAILVYNLVYVEKISFLVACRVETSEVTGYASFCCNHTK 305
LRRC8B_Homo     EQKDIIYRVYLKQIIVKVILFVLIITYVPYFLTHITLEIDCSVDVQAFTGYKRYQCVYSL 308
                          TM4                 IL2H1
LRRC8A_Homo     ATLFKILASFYISLVIFYGLICMYTLWWMLRRSLKKYSFESIREESSYSDIPDVKNDFAF 374
LRRC8A_Mus      ATLFKILASFYISLVIFYGLICMYTLWWMLRRSLKKYSFESIREESSYSDIPDVKNDFAF 374
LRRC8A_Gallus   ATLFKILASFYISLVVVYGLICMYTLWWMLRRSLKKYSFESIREESSYSDIPDVKNDFAF 374
LRRC8A_Xenopus  ATLFKILASFYISLVGFYGLVCVYTLWWMLRRSLKKYSFESIREESSYSDIPDVKNDFAF 373
LRRC8D_Homo     AYMLKKLLISYISIICVYGFICLYTLFWLFRIPLKEYSFEKVREESSFSDIPDVKNDFAF 418
LRRC8C_Homo     AHLFSKLSFCYLCFVSIYGLTCLYTLYWLFYRSLREYSFEYVRQETGIDDIPDVKNDFAF 372
LRRC8E_Homo     AHLFSKLAFCYISFVCIYGLTCIYTLYWLFHRPLKEYSFRSVREETGMGDIPDVKNDFAF 365
LRRC8B_Homo     AEIFKVLASFYVILVILYGLTSSYSLWWMLRSSLKQYSFEALREKSNYSDIPDVKNDFAF 368
                 IL2H2    IL2H3      IL2H4
LRRC8A_Homo     MLHLIDQYDPLYSKRFAVFLSEVSENKLRQLNLNNEWTLDKLRQRLTKNAQDKLELHLFM 434
LRRC8A_Mus      MLHLIDQYDPLYSKRFAVFLSEVSENKLRQLNLNNEWTLDKLRQRLTKNAQDKLELHLFM 434
LRRC8A_Gallus   MLHLIDQYDPLYSKRFAVFLSEVSENKLRQLNLNNEWTLEKLRQRITKNSQDKLELHLFM 434
LRRC8A_Xenopus  MLHLIDQYDPLYSKRFAVFLSEVSENKLRQLNLNHEWTLDKLRQRITKNSQEKLELHLFM 433
LRRC8D_Homo     LLHMVDQYDQLYSKRFGVFLSEVSENKLREISLNHEWTFEKLRQHISRNAQDKQELHLFM 478
LRRC8C_Homo     MLHMIDQYDPLYSKRFAVFLSEVSENKLKQLNLNNEWTPDKLRQKLQTNAHNRLELPLIM 432
LRRC8E_Homo     MLHLIDQYDSLYSKRFAVFLSEVSESRLKQLNLNHEWTPEKLRQKLQRNAAGRLELALCM 425
LRRC8B_Homo     ILHLADQYDPLYSKRFSIFLSEVSENKLKQINLNNEWTVEKLKSKLVKNAQDKIELHLFM 428
LRRC8A_Danio    MLHMIDQYDPLYSKRFAVFLSEVSENKLRQLNLNNEWTLEKLRQRITKNSQEKLELHLFM 420
```

Figure S1. Amino-acid sequence alignment of pore domains of LRRC8A orthologs and human LRRC8 paralogs, related to Figures 1–3.

The residues M1–M434 of the pore domain of HsLRRC8A are aligned with the equivalent residues of other orthologs and human LRRC8 paralogs. Residues of NT and TMs that are involved in polar interactions are highlighted in yellow. Secondary structures are marked for HsLRRC8A.

Figure S2

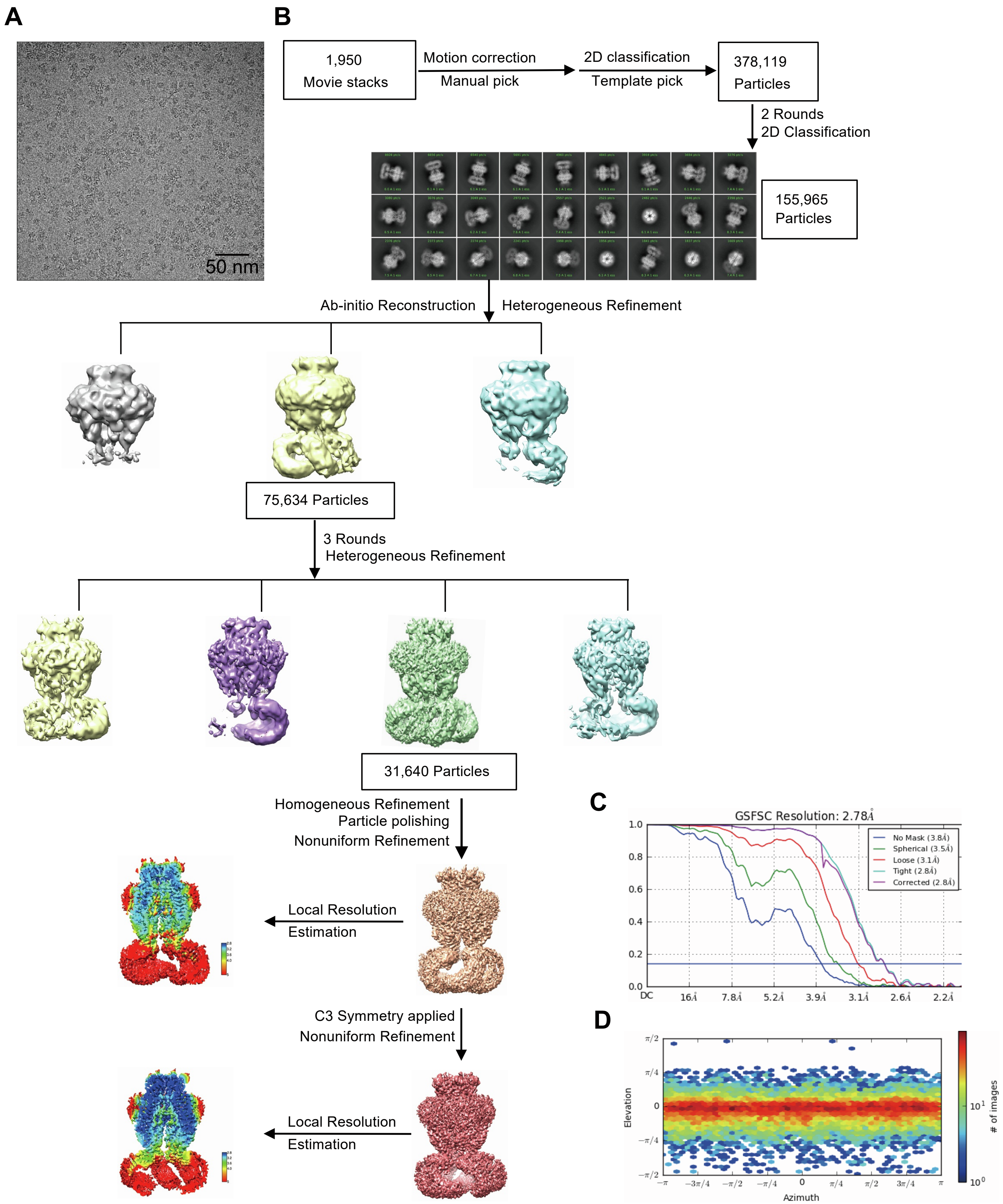

Figure S2. Reconstruction of cryo-EM structure of HsLRRC8A, related to Figures 1 and 2, and Table S1.

(A) Representative cryo-EM micrograph of dataset obtained at FEI Titan Krios. (B) Flowchart of HsLRRC8A reconstruction. Local resolution estimation is shown at the bottom panel. Flowchart of cryo-EM data processing of the HsLRRC8A structure, including particle picking, classification, and 3D refinement. (C) Fourier shell correlation (FSC) of the final 3D reconstruction following gold standard refinement. FSC curves are plotted before and after masking. (D) Angular distribution heatmap of particles used for the refinement.

Figure S3

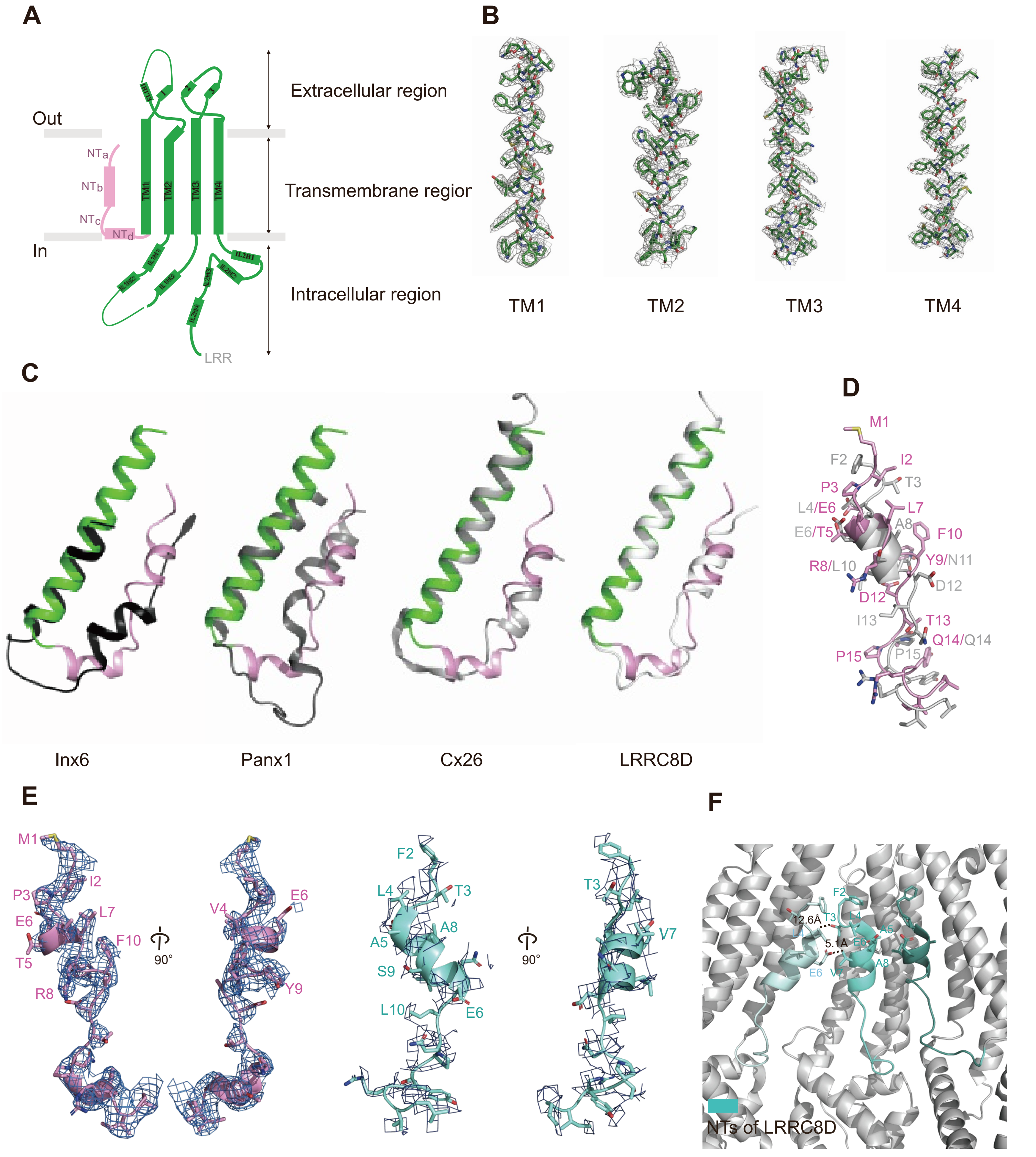

Figure S3. Subunit structure of HsLRRC8A, related to Figures 1 and 2.

(A) Schematic representation of the HsLRRC8A topology for the pore domain. (B) Cryo-EM density map (gray) for TMs. (C) Superimposition between HsLRRC8A NT and those of other large pore channels. PDB codes used in superimposition: INX6, 5H1Q[34]; Pannexin1, 6WBF[35]; Cx26, 2ZW3[33]; LRRC8D, 6M04[15]. (D) Superimposition of the HsLRRC8A NT (colored pink) and HsLRRC8D NT (colored grey), using the Cα atoms of the protomers. (E) Cryo-EM densities (contour level of each map is 2.0 σ) of NTs from a HsLRRC8A protomer (left) and a HsLRRC8D protomer (right), viewed from same orientations. (F) HsLRRC8D NTs and their local surroundings. No evident nonpolar and polar interactions were found between adjacent NTs.

Figure S4.

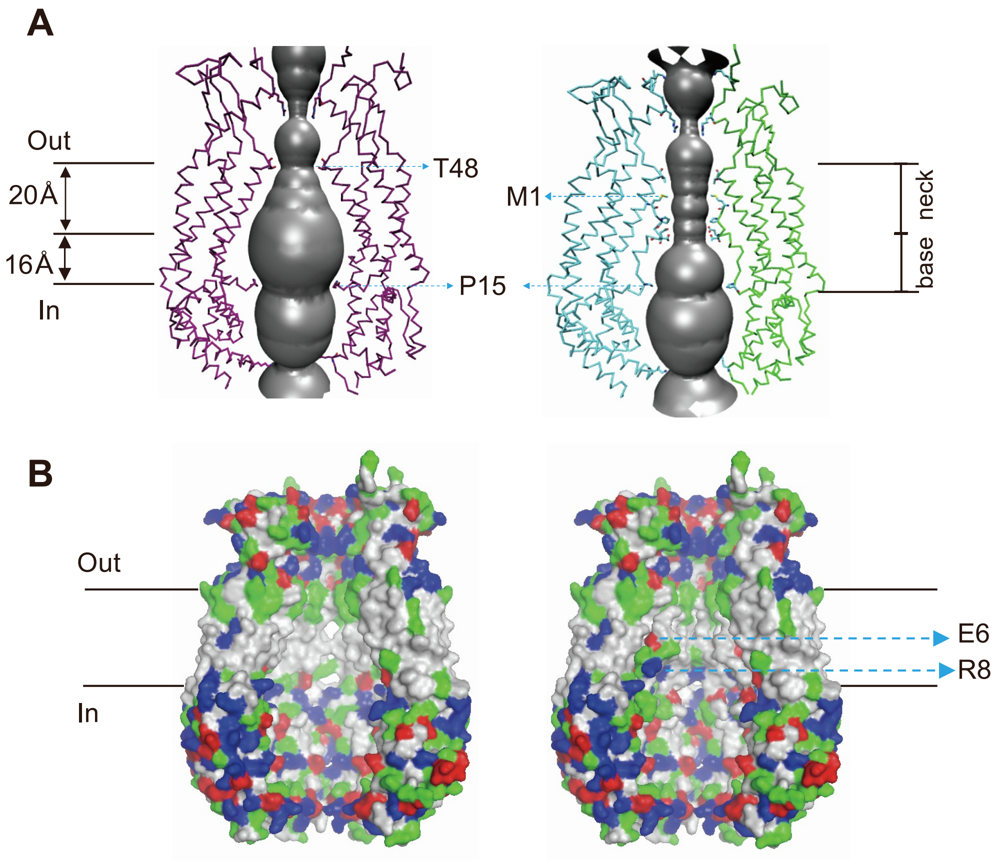

Figure S4. Permeation paths of LRRC8A channels containing unresolved NTs (left) and resolved NTs (right), related to Figures 1 and 2.

(A) Permeation paths relative to two opposite subunits. The neck and base have been marked. (B) Molecular surface of the permeation path viewed from the membrane. The surface is colored according to chemical properties of residues (hydrophobic, gray; hydrophilic, green; acidic, red; basic, blue). The PDB code is 5ZSU for HsLRRC8A containing the unsolved NTs. The two front subunits are removed for clarity.

Figure S5

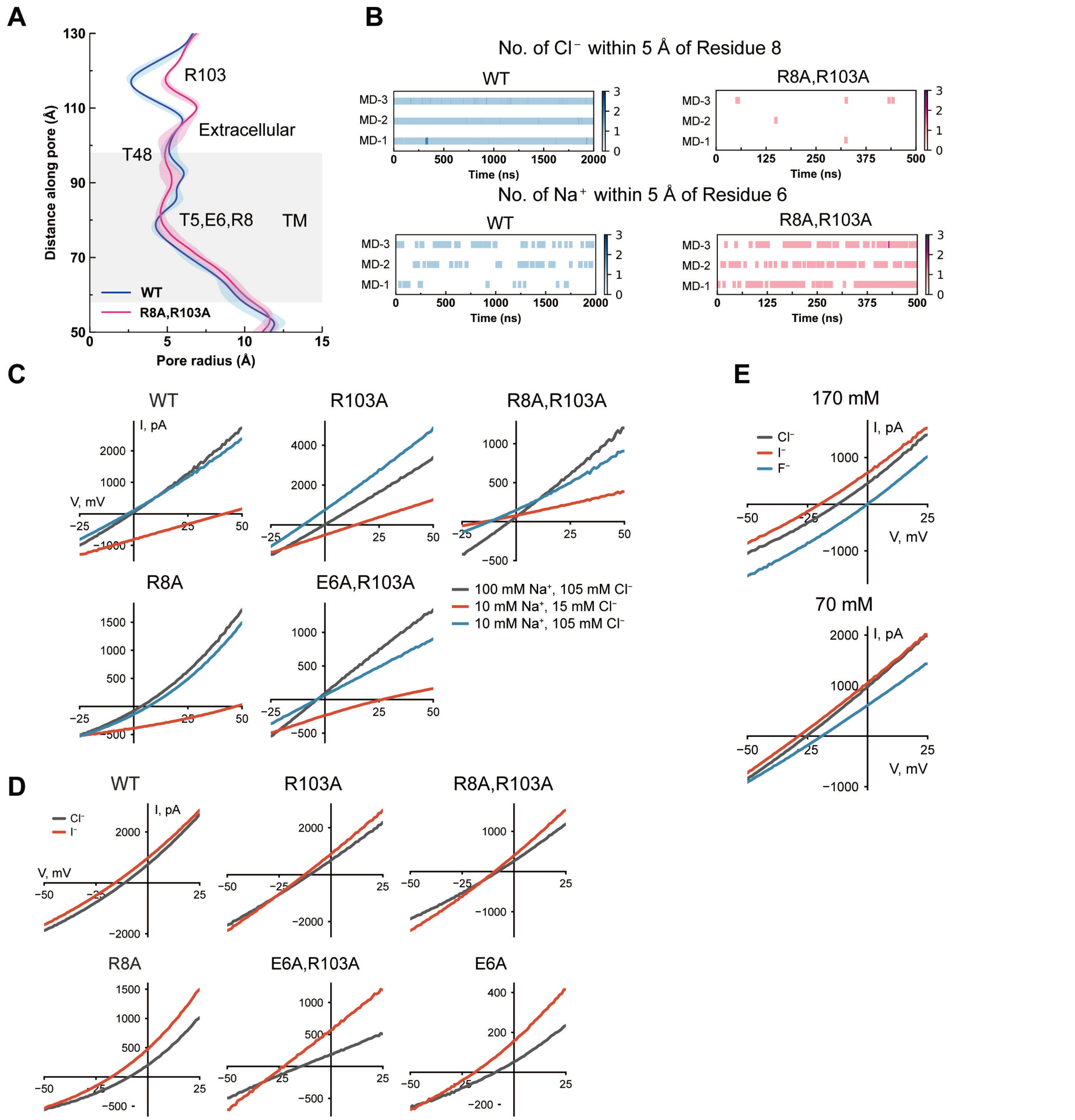

Figure S5. Influence of mutations on pore properties of LRRC8A channel as predicted by MD simulations (A and B) and tested by electrophysiology (C-E), related to Figure 3.
(A) Pore radius along the symmetry axis in WT (blue) and mutant (magenta) channels, respectively. The transmembrane segment of the pore domain is colored in grey. Radii were calculated from snapshots at 1-ns intervals in the last 1000-ns simulations. Data are shown as mean ± s.d. of three independent simulations for each system. (B) Number of Cl^- near residue 8 (top) and number of Na^+ near residue 6 (bottom) in WT (left) and the denoted mutant (right) channels during the course of simulations. (C) Averaged current traces from the recordings shown in Figures 3I and 3J, demonstrating the shifts in reversal potential between the high NaCl bath solution (gray), the low NaCl bath solution (red), and the low Na^+ (blue) bath solution. (D) Averaged current traces from the recordings shown in Figures 3K, 3L, and 3M, demonstrating the shifts in reversal potential between the NaCl (gray) and NaI (red) bath solutions. (E) Averaged current traces from the recordings shown in 4M and 4N, demonstrating the shifts in reversal potential between the NaCl- (gray), NaI- (red), and NaF-containing (blue) bath solutions.

Figure S6

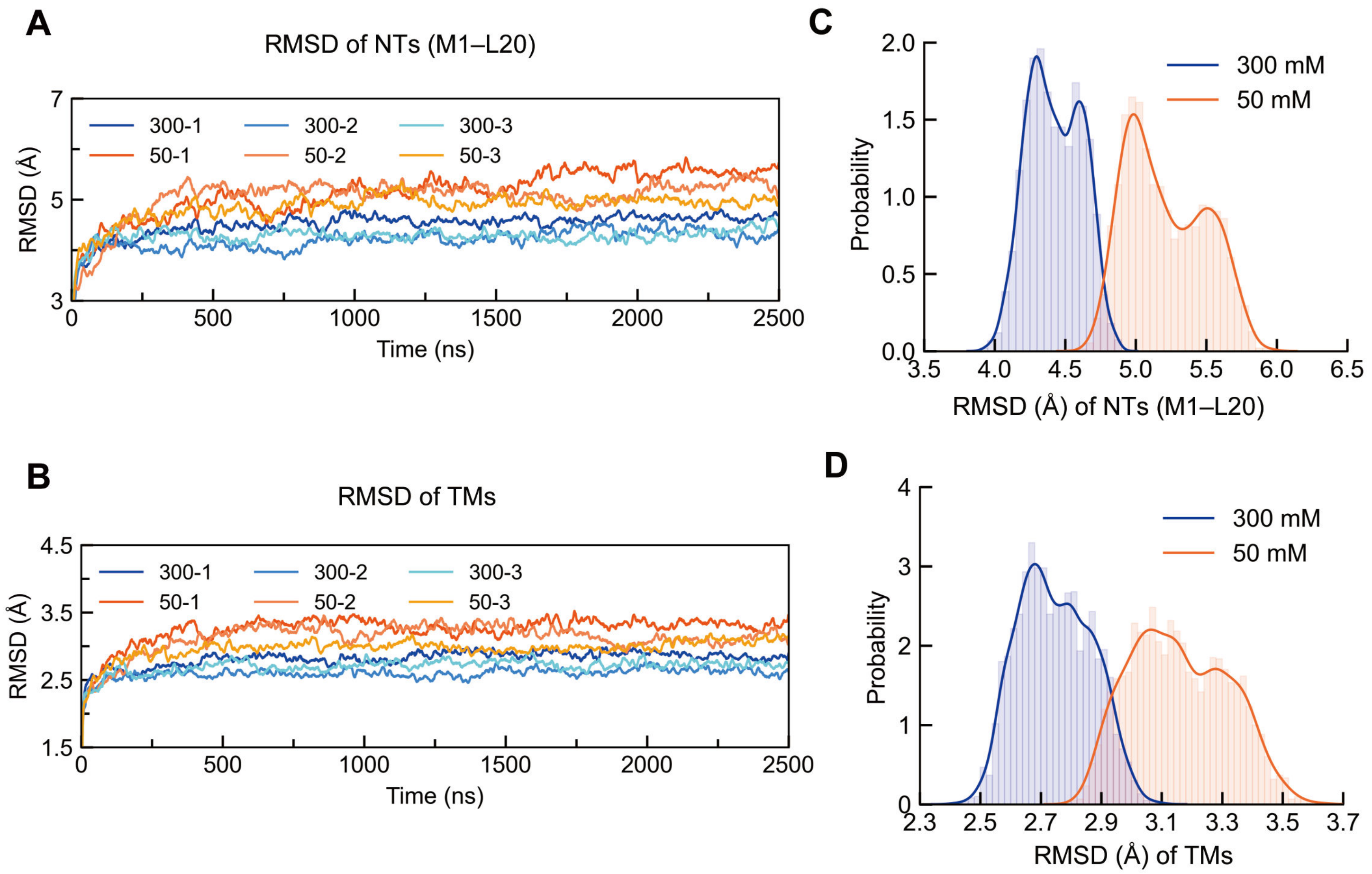

Figure S6. 2500-ns MD trajectories of RMSD values for residues of pore domain and their probability distributions generated from the last 1000-ns simulations, related to Figure 4.

The residue ranges of TMs were defined as: TM1, W23–T48; TM2, W120–K145; TM3, I259–N288; TM4, L314–M343. RMSD values of residues were calculated from their mainchain atoms sampled from snapshots at 100-ps intervals. (A and B) Trajectories of RMSD values for residues of NTs (A) or of TMs (B) in 2500-ns trajectories simulated at salt concentrations of 300 mM and 50 mM NaCl, respectively. Three independent simulations were performed at each salt concentration. (C and D) Probability distributions of RMSD values for residues of NTs (C) or of TMs (D) generated from the last 1000-ns trajectories.

Figure S7

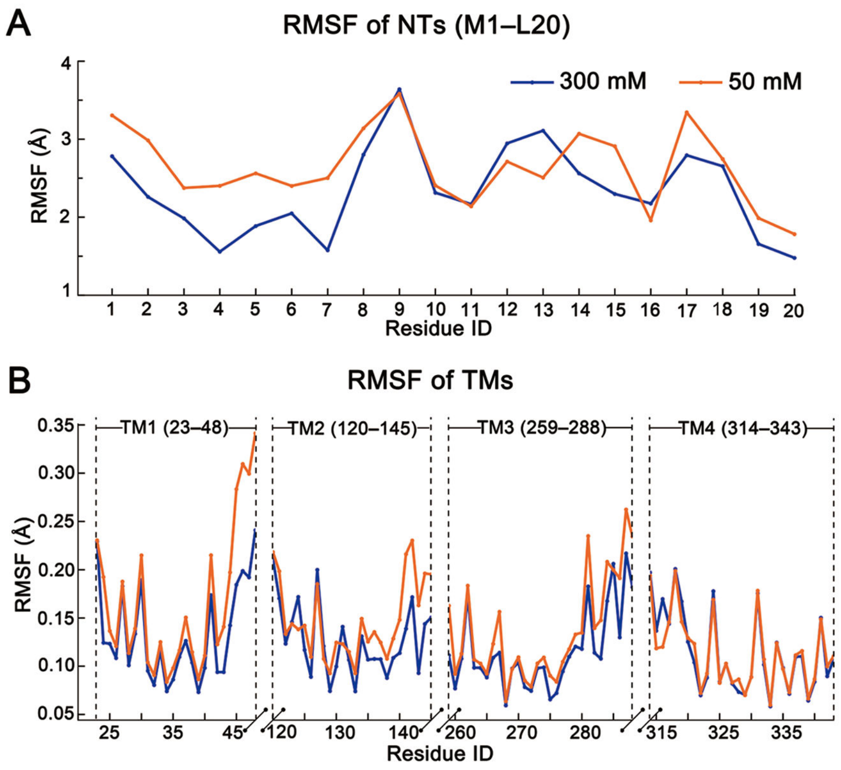

Figure S7. Root mean square fluctuations (RMSF) of each residue of NTs and TMs at 300 mM and 50 mM NaCl, related to Figure 4.

Root mean square fluctuations (RMSF) of each residue of NTs (A) and TMs (B) at 300 mM and 50 mM NaCl. Each subunit in a hexameric HsLRRC8A channel was treated equally. The RMSF value of each residue was calculated based on 18,000 sampled snapshots from all three parallel simulations, as the last 1000-ns snapshots at 1-ns intervals for each of the six subunits were used.

Figure S8

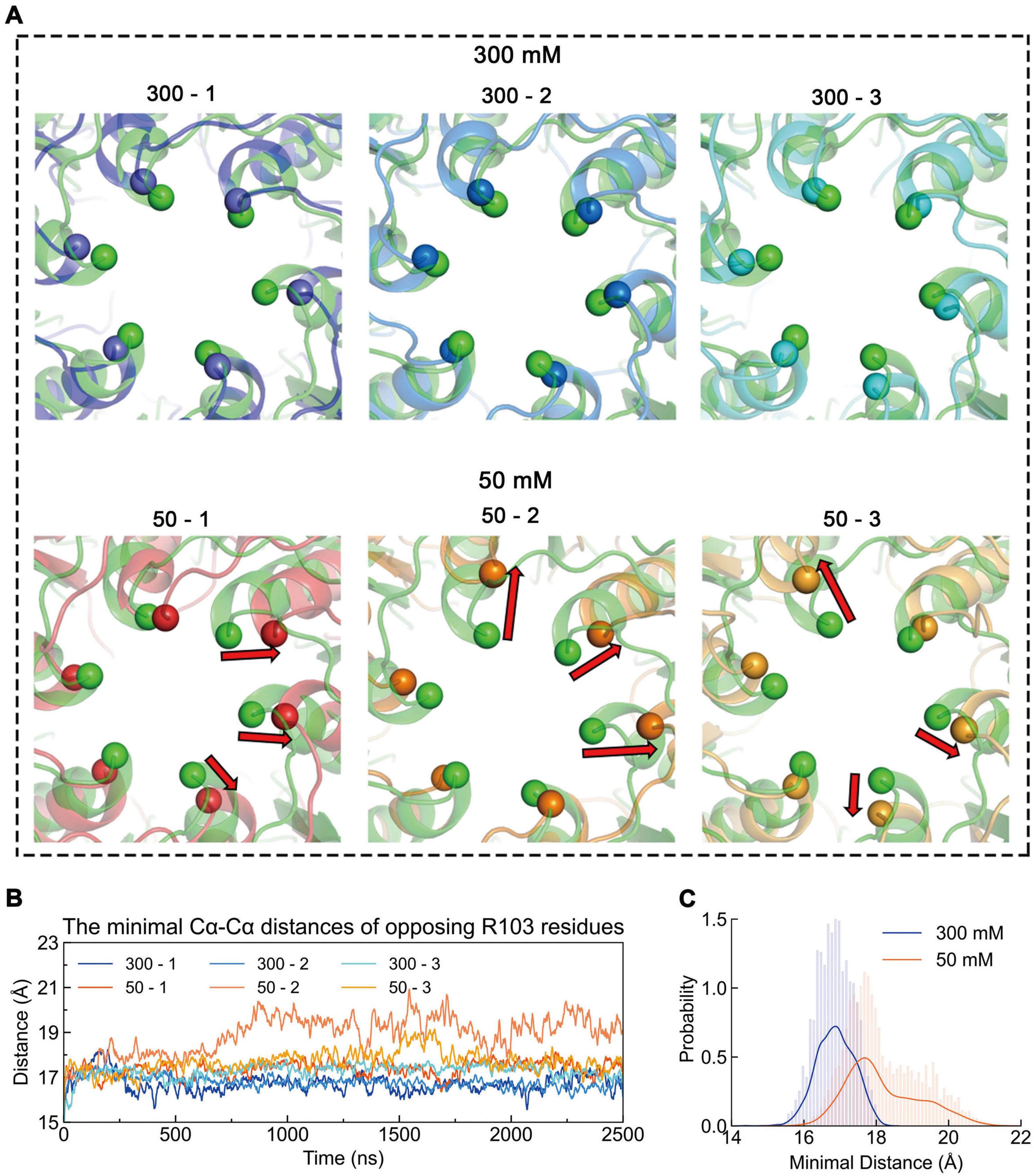

Figure S8. Radial movements of R103 in MD simulations conducted at 300 mM and 50 mM NaCl concentrations, related to Figure 4.

(A) Superposition of representative structures obtained at each salt concentration with the cryo-EM structure (colored in green) of HsLRRC8A, viewed from the top. Cα atoms of R103 residues were depicted as spheres. The red arrows indicate the radial dilation of Cα atoms of R103 residues at 50 mM NaCl. (B) The time evolution of minimal Cα-Cα distances between opposing R103 residues. (C) The probabilities of the Cα-Cα minimal distances calculated for the last 1000-ns simulations.

Figure S9

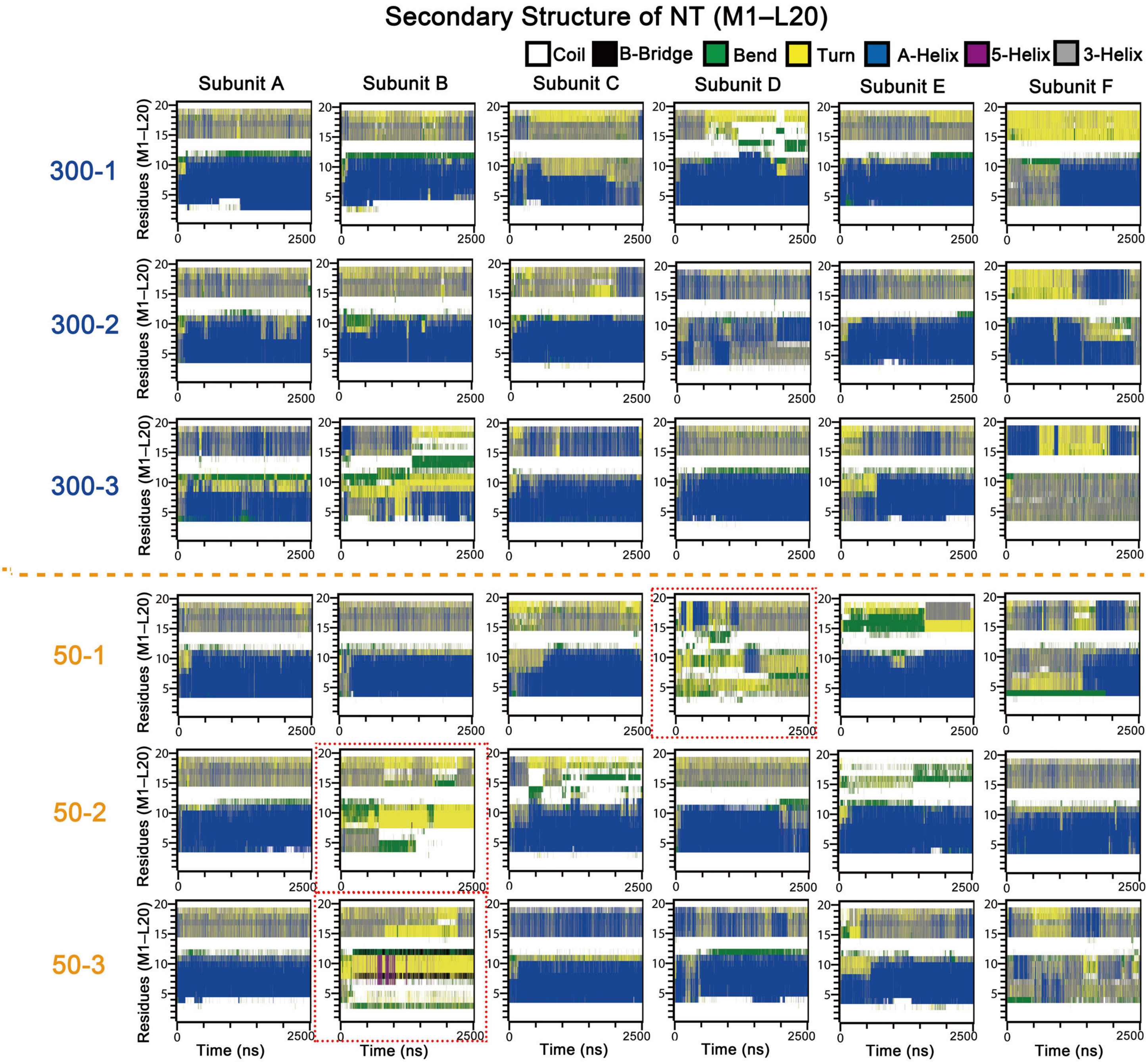

Figure S9. The changes in secondary structures of NT subsegments during the course of simulations, related to Figure 4.

The unwound subsegments at 50 mM NaCl are highlighted by red dashed boxes.

Table S1. Cryo-EM data collection, refinement, and validation statistics, related to Figure 1 and Figure S2.

**Data collection and processing**	
Microscope	FEI Titan Krios
Camera	K3
Voltage (kV)	300kV
Magnification	105K
Pixel size (Å)	0.52 (1.04)
Electron exposure (e–/Å^2)	~60
Defocus range (μm)	–1.0 to –2.2
Symmetry imposed	C3
Initial particle images (no.)	378,119
Final particle images (no.)	31,640
Map resolution (Å)	2.78
FSC threshold	0.143
**Refinement**	
Initial model used (PDB code)	6G9O
d model	3.2
dFSC model (0/0.143/0.5)	2.7/2.8/3.2
Map sharpening B factor (Å^2)	–78
Model composition	
Non-hydrogen atoms	36203
Protein residues	4385
B factor (Å^2)	304.66
R.m.s. deviations	
Bond lengths (Å)	0.009
Bond angles (°)	1.267
Validation	
MolProbity score	1.92
Clash Score	10.34
Poor rotamers (%)	0
Ramachandran plot	
Favored (%)	95.37
Allowed (%)	4.47
Disallowed (%)	0.16

Table S2. Cα-Cα distance between pairs of opposite residues that constitute the constriction sites along the permeation path, related to Figures 1 and 2.

**Distance (Å) between Cα atoms of opposite residues**	**R103**	**T48**	**M1 / P3**	**E6**	**P15**	**K235**
**HsLRRC8A**	14.7	16.8	22.7 / 17.1	19.9	28.5	24.5
**6G9O**	15.7	16.9	NA	NA	27.5	28.2
**6NZW**	15.5	18.3	NA	NA	28.7	27.7
**6NZZ**	16.1	18.3	NA	NA	32.4	31.9
**5ZSU**	16.2	18.4	NA	NA	NA	33.9
**6DJB**	15.3	18.8	NA	NA	35.5	31.0

Table S3. Buried surface area in the interface between two adjacent subunits of the pore domain, related to Figure 2. The extracellular, transmembrane and intracellular portions of pore domain are same as those in Figure 1.

**Buried surface (Å²)**	**HsLRRC8A**	**6G9O**	**5ZSU (loose/tight interface)**	**6DJB**	**6NZW**	**6NZZ**
**Total**	2180	1683	1701/1051	1668	1590	1537
**Extracellular segment**	920	915	850/805	860	887	920
**Transmembrane segment**	810	380	376/234	302	379	314
**Contribution of N-halves to transmembrane segment**	770	NA	NA	NA	NA	NA
**Contribution of C-halves to transmenbrane segment**	445	NA	NA	NA	NA	NA
**Intracellular segment**	490	328	464/NA	398	311	266
