# Supplementary material for: Structural insights into anion selectivity and activation mechanism of LRRC8 volume-regulated anion channels
Source: Cell Rep. 2023 Aug 6;42(8):112926. doi: 10.1016/j.celrep.2023.112926 (PMC10480491; doi:10.1016/j.celrep.2023.112926)
Supplement: Document S2. Article plus supplemental information [file mmc2.pdf]

Cell Reports

Article

Structural insights into anion selectivity and activation mechanism of LRRC8 volume-regulated anion channels

Graphical abstract

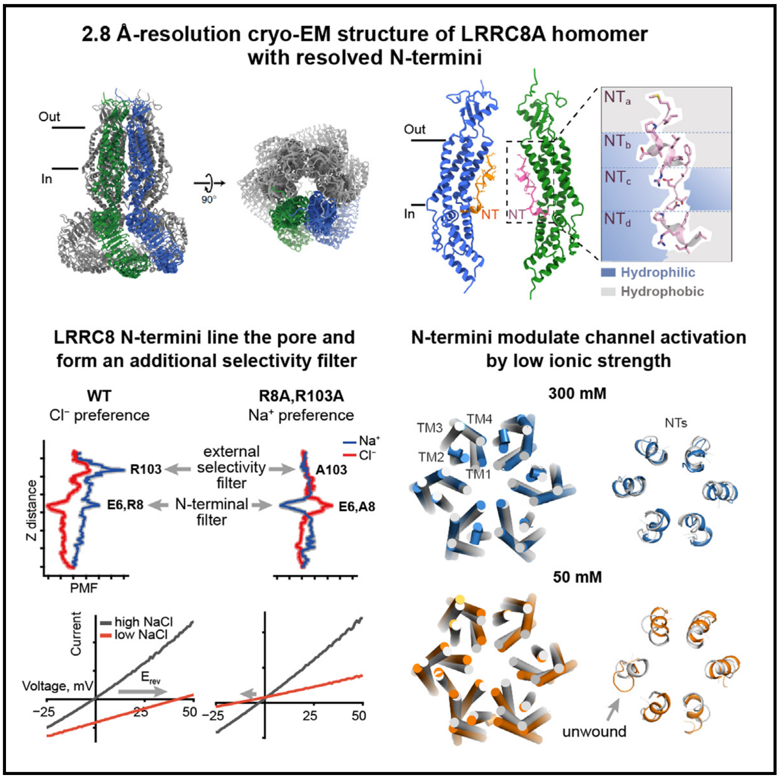

Highlights

- Cryo-EM structure of LRRC8A volume-regulated anion channel reveals back-folded N termini
- N termini form a selectivity filter in series with a second one at the external pore opening
- Molecular dynamics calculations suggest that low ionic strength partially unwinds N termini
- The unwound N termini may modulate channel activation by cell swelling

Authors

Heng Liu, Maya M. Polovitskaya, Linlin Yang, ..., Qiansen Zhang, Thomas J. Jentsch, Jun Liao

Correspondence

yanglin-89@zzu.edu.cn (L.Y.),
qszhang@bio.ecnu.edu.cn (Q.Z.),
jentsch@fmp-berlin.de (T.J.J.),
liaojun@shanghaitech.edu.cn (J.L.)

In brief

Liu et al. show that the N terminus of LRRC8A, resolved in their structure of a volume-regulated VRAC anion channel, inserts into the pore. It forms a second selectivity filter in series with an external one. Its partial unfolding under low ionic strength may trigger activation of VRAC by cell swelling.

Check for updates

Liu et al., 2023, Cell Reports *42*, 112926
August 29, 2023 © 2023 The Author(s).
https://doi.org/10.1016/j.celrep.2023.112926

CellPress

Cell Reports

CellPress
OPEN ACCESS

Article

Structural insights into anion selectivity and activation mechanism of LRRC8 volume-regulated anion channels

Heng Liu,[1,2,3,8] Maya M. Polovitskaya,[4,8] Linlin Yang,[5,*] Meiling Li,[5] Hongyue Li,[1,2,3] Zhen Han,[5] Jianguo Wu,[1,2,3] Qiansen Zhang,[6,*] Thomas J. Jentsch,[4,7,*] and Jun Liao[1,2,3,9,*]
[1]School of Life Science and Technology, ShanghaiTech University, Shanghai 201210, China
[2]Shanghai Institute of Biochemistry and Cell Biology, Center for Excellence in Molecular Cell Science, Chinese Academy of Sciences, Shanghai 200031, China
[3]University of Chinese Academy of Sciences, Beijing 100049, China
[4]Leibniz-Forschungsinstitut für Molekulare Pharmakologie (FMP) and Max-Delbrück-Centrum für Molekulare Medizin (MDC), 13125 Berlin, Germany
[5]Department of Pharmacology, School of Basic Medical Sciences, Zhengzhou University, Zhengzhou, Henan 45001, China
[6]Shanghai Key Laboratory of Regulatory Biology, Institute of Biomedical Sciences, School of Life Sciences, East China Normal University, Shanghai, China
[7]Cluster of Excellence NeuroCure, Charité Universitätsmedizin Berlin, Berlin, Germany
[8]These authors contributed equally
[9]Lead contact
*Correspondence: yanglin-89@zzu.edu.cn (L.Y.), qszhang@bio.ecnu.edu.cn (Q.Z.), jentsch@fmp-berlin.de (T.J.J.), liaojun@shanghaitech.edu.cn (J.L.)
https://doi.org/10.1016/j.celrep.2023.112926

SUMMARY

Volume-regulated anion channels (VRACs) are hexamers of LRRC8 proteins that are crucial for cell volume regulation. N termini (NTs) of the obligatory LRRC8A subunit modulate VRACs activation and ion selectivity, but the underlying mechanisms remain poorly understood. Here, we report a 2.8-Å cryo-electron microscopy structure of human LRRC8A that displays well-resolved NTs. Amino-terminal halves of NTs fold back into the pore and constrict the permeation path, thereby determining ion selectivity together with an extracellular selectivity filter with which it works in series. They also interact with pore-surrounding helices and support their compact arrangement. The C-terminal halves of NTs interact with intracellular loops that are crucial for channel activation. Molecular dynamics simulations indicate that low ionic strength increases NT mobility and expands the radial distance between pore-surrounding helices. Our work suggests an unusual pore architecture with two selectivity filters in series and a mechanism for VRAC activation by cell swelling.

INTRODUCTION

Volume-regulated anion channels (VRACs) are important players in cell volume regulation.[1,2] Several decades after the first description of VRAC currents, leucine-rich repeat-containing 8 (LRRC8) proteins have been identified as constituting VRACs.[3,4] The mammalian LRRC8 gene family encodes five paralogs, LRRC8A to LRRC8E (Figure S1).[5] Physiological VRACs consist of obligatory LRRC8A subunits that are associated with at least another paralog to form functional channels with distinct composition-dependent permeation and activation properties.[3,6,7]

LRRC8 channels are selective for inorganic anions but also permeate various small organic substrates.[1,3,4,6,8–10] An LRRC8 subunit consists of an amino terminus (NT), four transmembrane helices, and a carboxyterminal LRR (leucine-rich repeat) domain. Recent cryo-EM studies of LRRC8A, LRRC8D, and LRRC8A/C channels reveal a hexameric assembly of LRRC8 subunits around the central axis of symmetry that defines the ion conduction pore.[11–18] Arginine 103 (R103) residues in the first extracellular loop (EL1) of LRRC8A, together with positionally equivalent uncharged residue of other LRRC8 subunits in heteromeric channels, constrict the extracellular portion of the pore and form a selectivity filter in LRRC8 channels.[11–17] However, channels in which R103 of LRRC8A has been replaced by uncharged residues remain selective for anions,[11,13,19] suggesting that additional elements are involved in anion perm-selectivity.[11–15] Indeed, mutations in NTs of LRRC8A and LRRC8C subunits have been associated with altered anion permeation of VRACs.[11,20]

LRRC8 channels can be activated by low ionic strength and cell swelling.[21–23] Functional studies have implicated the NT, the first intracellular and extracellular loops (IL1 and EL1, respectively), cytoplasmic LRR domains, and oligomerization

This is an open access article under the CC BY license (http://creativecommons.org/licenses/by/4.0/).

stoichiometry in the regulation of channel activation.[16–20,24–28] Nonetheless, the mechanism of how low ionic strength/cell swelling activates LRRC8/VRAC channels remains unclear. This paucity of knowledge is due in part to a lack of LRRC8/VRAC channel structures in both closed and open states, which might be obtained at high and low ionic strength, respectively.

Work on homomeric LRRC8A and heteromeric LRRC8A/C channels has produced so far only structures with unresolved NTs.[11–13,17,18,29] This is unfortunate since NTs were suggested to fold into the pore of the channel and have important functional effects.[20] Although a cryo-EM structure of human LRRC8D (hLRRC8D) revealed an ordered NT in the pore,[15] the low-resolution (≥4.36 Å resolution) density map and poor side-chain densities make unambiguous identification of residues difficult. In addition, the differences in the NT sequences (Figure S1) of LRRC8A and D paralogs suggest they may adopt different structures and exert composition-dependent influences on LRRC8/VRAC activities. Here, we present the 2.8-Å cryo-EM structure of homomeric human LRRC8A (HsLRRC8A) channels that for the first time shows NTs resolved at a local resolution of 3.0–3.2 Å. NTs of HsLRRC8A, adopting an unusual fold, interact with pore-surrounding TMs and ILs. The structure, together with molecular dynamics (MD) simulations and electrophysiological analyses, provides a framework for understanding the role of NTs in determining anion selectivity and activation of LRRC8/VRAC channels.

RESULTS AND DISCUSSION

High-resolution HsLRRC8A structure displays well-resolved NTs

The HsLRRC8A protein was heterologously overexpressed in HEK293S GnTI$^-$ cells and purified at high NaCl concentration (≥300 mM, see STAR Methods). Using these conditions, we obtained a high-resolution cryo-EM density map of HsLRRC8A that revealed atomic details of ordered NTs in the channel's pore (Figures 1 and S2).

The cryo-EM density map of HsLRRC8A achieved an overall resolution of 2.8 Å with imposed C3 symmetry (Figures 1A–1C, S2, and S3 and Table S1). While local resolution is as high as 2.7 Å in most of the pore domain, it drops to 4.5 Å in LRR domains that are more flexible[11–14,16] (Figure S2). The final model was built for residues M1–W411 of the pore domain, with the exception of disordered residues F69–D91 in EL1 and residues V177–G230 of the intracellular loop IL1. The model for the LRR domain (T412–E808) was docked into the density map using the crystal structure of this region (PDB: 6FNW).[13]

Resembling previous structures from mouse[13,14] and human[11,12] LRRC8A, the present HsLRRC8A pore domain is assembled as a trimer of dimers around the axis of C3 symmetry along the ion conduction path (Figures 1A–1C and S2). In the pore domain, the pairs of subunits in an asymmetric structural unit have the root-mean-square deviation (RMSD) of 0.36 Å for 330 super-imposed pairs of Cα atoms (Figure 1D), suggesting nearly identical structures. Putative lipid molecules were identified at inter-subunit gaps (Figure 1A), akin to findings obtained for nanodisc-embedded mouse LRRC8A.[14]

NTs support a tightly packed pore domain and constrict the permeation path

The pore, ∼110 Å long, has an extracellular, a main transmembrane, and an intracellular portion (Figures 1A, 1B, S3A, and S3B). Concordant with an accessibility analysis,[20] the NTs, resolved at 3.0- to 3.2-Å resolution, locate at the inner surface of the pore domain and span nearly the entire width of the membrane (Figures 1E–1J). NTs of LRRC8 channels were thought to bear structural resemblance to those of innexins, connexins, and pannexins.[5,32,33] In those channels, NTs adopt a typical fold of either a regular α helix followed by a loop or a regular α helix sandwiched by two loops[34–37] (Figure S3C). However, the NT (M1–L20) of HsLRRC8A channels can be partitioned into four subsegments, which we named NTa (loop, M1–P3), NTb (3_{10} helix, V4–F10), NTc (loop, A11–Q14), and NTd (3_{10} helix, P15–L20) (Figures 1E, 1F, S3A, and S3C). While NTb and NTd are amphipathic, NTa is hydrophobic and NTc hydrophilic (Figure 1F). These segments are differently oriented relative to the membrane bilayer (Figures 1E, 1H, and S3C): NTd lies nearly parallel to the bilayer and proximal to cytoplasm, NTc abruptly bends with an ∼90° angle, followed by NTb tilted at ∼60° angle and NTa bent again at ∼90° angle from the membrane plane. HsLRRC8A NT displays marked differences from HsLRRC8D NT (Figures S3D–S3F): the RMSD is 3.30 Å for super-imposed 19 pairs of Cα atoms (residues 2–20). Whereas HsLRRC8A NTs participate in interactions between the subunits (Figure 2), HsLRRC8D NTs contribute little to oligomerization[15] (Figure S3F).

The extracellular and intracellular segments of the pore domain are most tightly constricted at residues R103 and K235, respectively (Figures 1G, 1H, and 1J). Compared to the other published LRRC8A structures,[11–14] the present pore domain has the shortest distances between the opposing Cα atoms of R103 or K235 residues (Table S2), suggesting it is the most tightly packed. In structures with unresolved NTs, the oligomer is largely stabilized by close subunit interactions at the extracellular segment, with the buried surface area between the adjacent subunits being more than the sum of those in other segments (Table S3). In the present structure, however, the oligomer is further stabilized by interactions involving NTs (Figures 2A–2F and Table S3): hydrophobic residues of NTa, NTb, and TM1 and TM2 of one subunit, and hydrophobic residues of NTa and TM1 of the adjacent subunit engage in non-polar interactions (Figure 2C), while E6 and Y9 of NTb in one subunit and T5 and R8 of NTb in the adjacent subunit, respectively, form H-bonds (Figure 2D).

In LRRC8A structures with unresolved NTs, the transmembrane permeation path is lined by residues of TM1 throughout its length and additionally by residues of TM2 on the intracellular half[11–14,16] (Figures 1J, S4A, and S4B). In the present structure with resolved NTs, the permeation path is partitioned into a neck and a base (Figures 1G, 1H, 1J, S4A, and S4B). The neck, with an average diameter of approximately 11.2 Å, is lined by residues of the N-halves of the NTs and by residues of the extracellular halves of TM1. The base is lined by residues of the NT C-halves and by residues of TM1s and TM2s toward the intracellular side, where the base reaches a diameter of approximately 21.0 Å. The base, with the diameter gradually

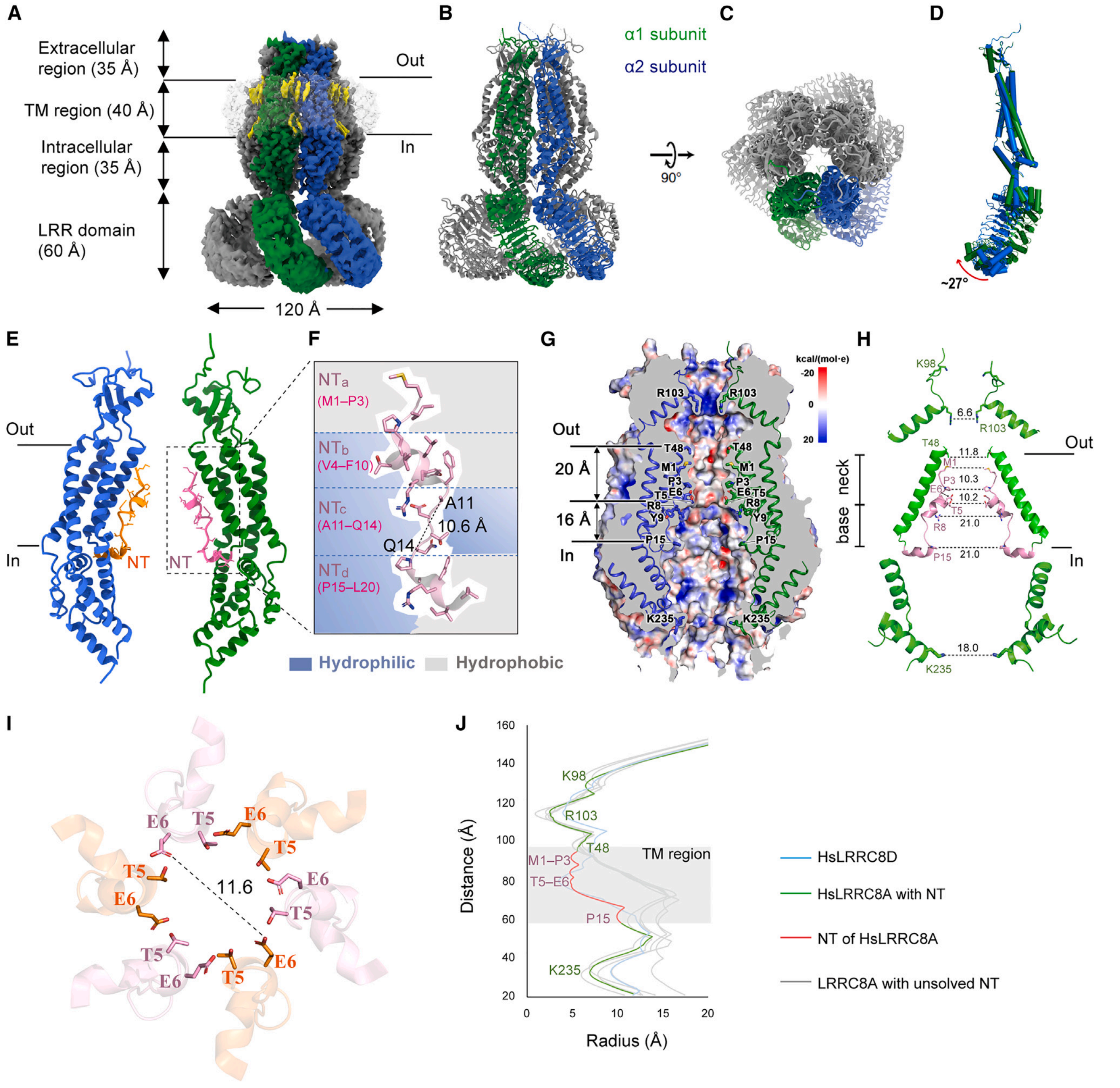

Figure 1. NTs constrict permeation path

(A) Cryo-EM density map of HsLRRC8A. Solid lines mark the membrane boundaries. Yellow densities represent putative lipid molecules.

(B and C) HsLRRC8A structure, viewed parallel to the membrane (B) and from the extracellular side (C).

(D) Superimposition of the two LRRC8A subunits in an asymmetric structural unit, using the Cα atoms of the transmembrane helices. The structures are depicted in cylinder representations.

(E) Side view of the pore domain of HsLRRC8A. NTs in opposite subunits are highlighted in orange and pink, respectively.

(F) Close-up view of NT; its four subsegments have different chemical properties: hydrophilic parts are shown in blue, and hydrophobic parts are shown in gray.

(G) Electrostatic potential of the channel pore mapped on the molecular surface in a vertical cross-section view. Important pore-lining residues are depicted as sticks. The electrostatic potential was calculated by solving the Poisson-Boltzmann equation at 300 mM NaCl using the PBEQ Solver module on CHARMM-GUI server.[30]

(H) Zoomed-in view of pore-lining NT and TM1. The neck (height ~20.0 Å) and base (height ~16.0 Å) of the permeation path have been marked.

(I) The pore at T5/E6 viewed from the extracellular side. The distance between two opposing OE1 atoms was calculated by the HOLE[31] program.

(J) The pore radius along the symmetry axis. The PDB codes for LRRC8A channels: 6G9O,[13] 6NZW,[14] 6NZZ,[14] 5ZSU,[12] and 6DJB.[11] The PDB code for LRRC8D: 6M04.[15] Only two opposite subunits viewed parallel to the membrane are shown for clarity in (E), (G), and (H).

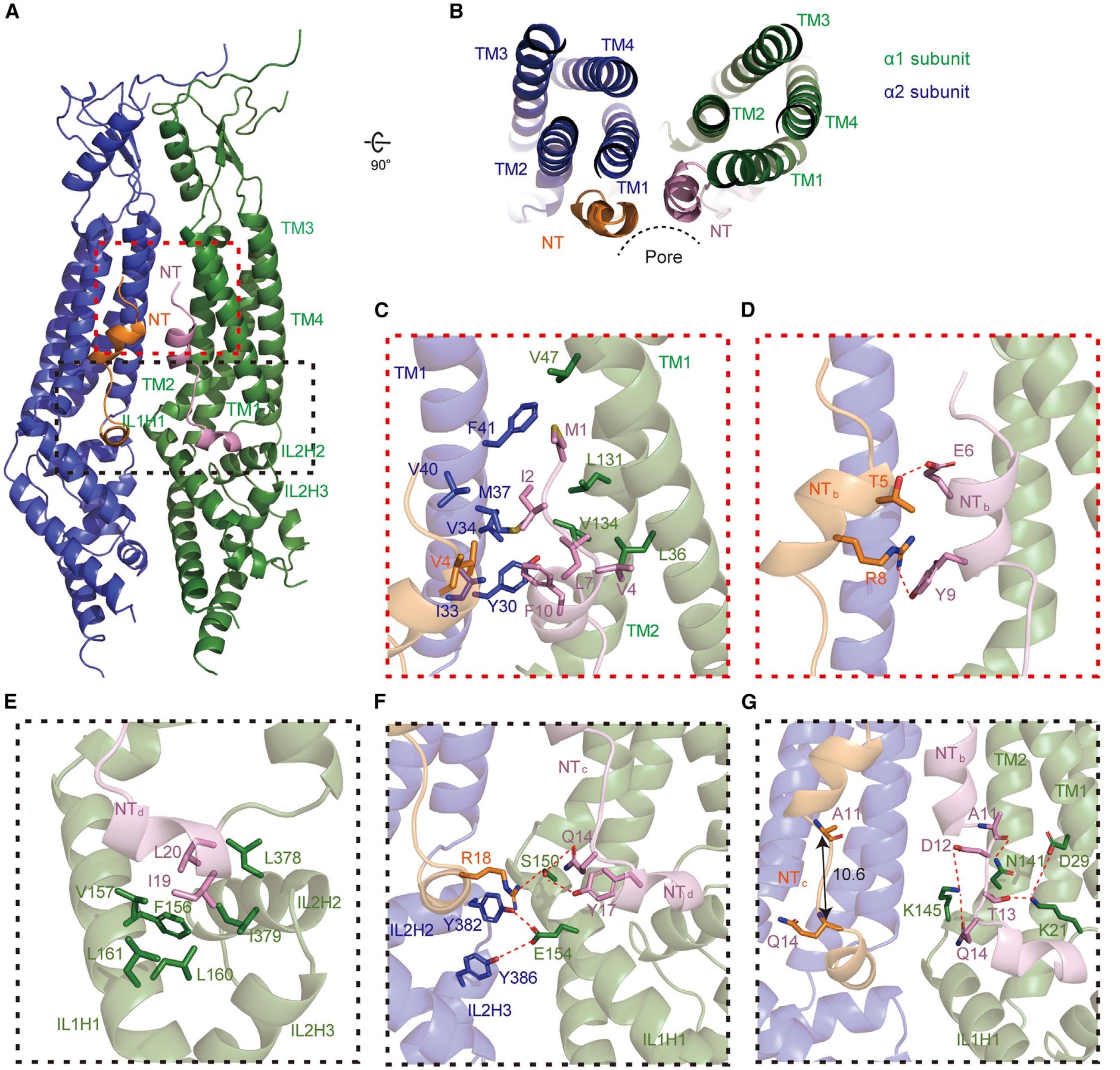

Figure 2. Ordered NTs stabilize a compact assembly of the pore domain

(A and B) Two adjacent LRRC8A pore domains in an asymmetric structural unit, viewed from the membrane plane (A) and from the top (B). The dashed boxes in (A) highlight the N-terminal halves (red box) and C-terminal haves (dark gray box) of two adjacent NTs and their interacting secondary structures.

(C) Non-polar interactions involving NTa, NTb, and TMs.

(D) Polar interactions involving adjacent NTb subsegments.

(E and F) Non-polar (E) and polar (F) interactions involving NTd and intracellular loops IL1 and IL2.

(G) Polar interactions involving NTc and TMs in same subunit.

increasing to 21.0 Å, is lined by residues of the NT C-halves and by residues of TM1s and TM2s toward the intracellular side. The narrowest portion of the neck is situated at residues M1, P3, and T5, where it displays diameters of about 10.3, 10.3, and 10.2 Å, respectively (Figures 1H and S4A). Further toward the cytoplasmic opening, the side chains of charged E6 and R8 residues protrude into the pathway. The opposing carboxylate and guanidinium groups of these residues display the shortest distances of approximately 11.6 and 21.0 Å, respectively (Figures 1H and 1J). Although the narrowest portion of the neck is wide enough for the passage of hydrated halide anions (~7.0 Å), it is narrower than the size (11.0–13.0 Å) required for the experimentally determined passage of larger anions and molecules in native VRACs.[38,39] This suggests

that the pore may be in a partially closed state and undergoes further dilation when channels are fully opened.

NTs interact with intracellular loops that are crucial for channel activation

The C half of NTs consist of an amphipathic NTd and a hydrophilic NTc (Figures 1F and 2E–2G). NTd engages intracellular loops IL1 and IL2 by non-polar and polar interactions at the membrane-cytoplasm interface (Figures 2E and 2F). The non-polar interactions involve hydrophobic residues on the hydrophobic face of NTd and of IL1H1 and IL2H2 in the same subunit (Figure 2E). The polar interactions involve hydrophilic residues of NTc, hydrophilic residues on the hydrophilic face of NTd and of IL1H1 of one subunit, and R18 and hydrophilic residues of IL2H2 and IL2H3 of the neighboring subunit (Figure 2F). Since these types of interactions are influenced by ionic strength,[40] conformations of ILs and NTd may be affected by ionic strength, a parameter known to modulate VRAC/LRRC8 gating.[10,19,21–24]

NTc is stretched: the Cα-Cα distance is 10.6 Å between A11 and Q14 (Figures 1F and 2G). The insertion of an arbitrary, flexible, six-residue fragment C-terminally to Q14 of both LRRC8A and LRRC8C abolished LRRC8A/C activity but not its trafficking to the plasma membrane,[20] suggesting that a stretched NTc is crucial for channel activation. Because the hydrophilic NTc engages TM1 and TM2 of the same subunit by polar interactions (Figure 2G), movements of NTc may induce changes in TM1 and TM2.

Serial selectivity filters determine ion selectivity

The role of R103 of LRRC8A in anion permeation has been extensively studied,[11,13] although only the LRRC8A and LRRC8B isoforms display arginine at this position. The mechanism by which the NT of LRRC8A influences ion permeation has remained largely unclear. E6 and R8 of NTb are the only charged residues protruding into the transmembrane portion of permeation path (Figures 1G–1J and 2D). In addition, these residues are conserved across most LRRC8 paralogs and orthologs (Figure S1). We performed MD simulations and potential of mean force (PMF) calculations to define the free-energy landscape of Na^+ and Cl^- permeation using the HsLRRC8A cryo-EM structure as the initial model (Figures 3A–3E, S5A, and S5B).

PMF calculations revealed two distinct free-energy maxima relevant to Na^+ permeation (Figure 3A). Consistent with other studies,[13,41] the highest PMF barrier is located at the extracellular constriction ($z \sim -1.3$ nm) constituted by six R103 residues (Figures 3A and S5A). Notably, a PMF barrier to Cl^- permeation is also conferred by this constriction (Figure 3A). However, the peak Cl^- barrier is about 8.2 kcal/mol lower than that for Na^+, suggesting that this constriction primarily impedes Na^+ passage. The site ($z \sim -5.2$ nm) at the NTs containing residues E6 and R8 generates the second highest PMF barrier to Na^+ permeation (Figures 3A and S5A). Importantly, an energy well for Cl^- (~ -6.1 kcal/mol) occurs at this site (Figure 3A), suggesting that it may attract anions as in other anion-selective channels or transporters.[42–44] MD simulations predicted that Cl^- accumulates at this site (Figures 3F and S5B). Intriguingly, different from the wide separation between E6 and R8 in cryo-EM structure (Figures 1H and 2D), the side chain of E6 in one subunit engages R8 in an adjacent subunit in MD simulations (Figures 3F and 3H). Consequently, negatively charged E6 and positively charged R8 collectively influence the local electrostatics at this segment of the pore, thereby affecting the local concentration of cations and anions and their passage (Figure S5B).

To better understand how E6 and R8 influence Na^+ and Cl^- permeation, we performed PMF calculations for homomeric LRRC8A channels with an alanine substitution of either residue or in combination with an alanine substitution of R103 (Figures 3B–3E). The alanine substitution of six R8 residues in LRRC8A (R8A) mutant reverses the energy landscape at this site for Na^+ and Cl^- permeation (Figure 3B): an energy barrier for Cl^- arises, while an energy well occurs for Na^+. However, the peak Na^+ barrier at R103 renders the R8A mutant still impermeable to Na^+. The LRRC8A (R8A, R103A) mutant is predicted to not only permeate but even favor Na^+ (Figures 3C and 3D). MD simulations also suggest an enrichment of Na^+ near the negatively charged E6 (Figures 3G and S5B). Intriguingly, the alanine substitutions of E6 and R103 remove the Na^+ barrier at R103 and slightly reduce the depth of Cl^- energy well for the LRRC8A (E6A, R103A) mutant (Figure 3E). These findings collectively indicate that LRRC8A (E6A, R103A) mutant is less Cl^- selective than the wild-type channel.

We ascertained some predictions from MD by electrophysiology. Because homomeric LRRC8A channels yield very small currents with non-physiological characteristics,[41] we examined physiological LRRC8A/C heteromeric channels. LRRC8A displays R103 and LRRC8C L105 at the extracellular constriction, while both subunits contain E6 and R8 in the transmembrane portion of permeation path (Figure S1). We mutated both E6 and R8 residues in either subunit (Figures 3I, 3J, and S5C). Alanine substitution of E6 decreased the whole-cell current density (Figure 3J). We found marked changes in ion selectivity (Figure 3I), an intrinsic channel property that unlike current amplitudes is independent of expression levels. We believe that the observed changes in current densities of E6A also reflect single-channel properties. This is because qualitatively similar changes in I^-/Cl^- permeability ratio and current amplitudes caused by the E6C mutation can be acutely rescued to wild-type (WT) levels by reconstituting a negative charge through cysteine modification.[20] As predicted by MD results (Figure 3C), and as described previously,[13,41] neutralizing R103 significantly increased HsLRRC8A/C channel's Na^+ permeability as indicated by a shift in the reversal potential. The LRRC8A/C (R8A/R8A, R103A/L105A) double mutant, predicted to be attractive for Na^+ and repulsive for Cl^- (Figure 3D), showed strikingly increased Na^+ over Cl^- preference (Figure 3I). As expected (Figure 3E), LRRC8A/C channels carrying the (E6A/E6A, R103A/L105A) mutations displayed moderately increased Cl^- preference, compared to the (R103A/L105A) mutant (Figure 3I).

The anion selectivity of LRRC8/VRACs conforms to an Eisenmann type I sequence ($SCN^- > I^- > NO_3^- > Br^- > Cl^- > F^-$).[1,8,9] Consistent with our previous observations,[20] both E6A and R8A mutations increased the I^-/Cl^- permeability ratio, while R103A had a negligible effect (Figures 3K, 3L, and S5D).

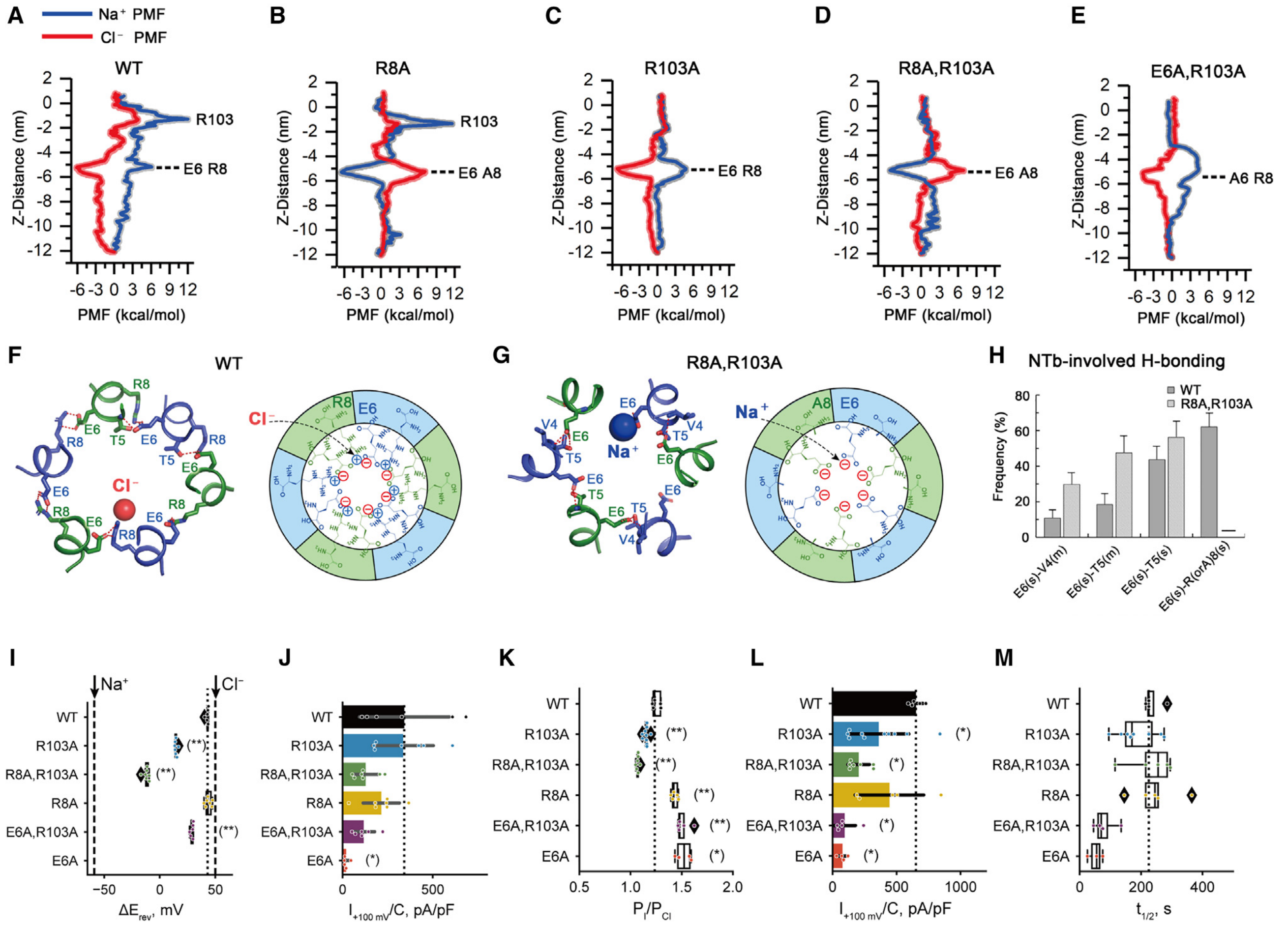

Figure 3. Charged residues of NTs and the first extracellular loops determine the ion selectivity

(A–E) Potential of mean force (PMF) describing the free-energy landscape (ΔG, kcal/mol) experienced by Na^+ ions (blue line) and Cl^- ions (red line) permeating the pore domain of LRRC8A (A), $LRRC8A^{R8A}$ (B), $LRRC8A^{R103A}$ (C), $LRRC8A^{R8A,\ R103A}$ (D), and $LRRC8A^{E6A,\ R103A}$ (E).

(F and G) Cl^- binding to E6/R8 pairs in wild-type HsLRRC8A (F) and Na^+ binding to E6 in $HsLRRC8A^{R8A,\ R103A}$ mutant (G) in a representative snapshot of MD trajectories (left) and its schematic representation (right). In an asymmetric unit, the side chain of E6 in one subunit and the side chain of R8 or T5 in the neighboring subunit form H-bonds. Cl^- and Na^+ are depicted by red and blue spheres, respectively. H-bonds are shown as red dashed lines.

(H) Frequency values of main-chain (m) and side-chain (s) H-bond interactions involving E6 and R8 (or A8) in WT channel and in the (R8A, R103A) mutant. The higher the value is, the more stable is a particular H-bond interaction. All data are mean ± SD of three independent simulations.

(I) Shifts in reversal potentials (ΔE_{rev}) of heteromeric LRRC8A/C channels upon reduction of NaCl in the bath, with both subunits either WT (black) or carrying the denoted (or equivalent) mutations in both LRRC8A and LRRC8C. E_{rev} for LRRC8A/C (E6A/E6A) could not be reliably measured under the given ionic conditions. The dotted line reflects the mean shift in E_{rev} for WT. Boxes show the median and the quartiles of the distribution. Expected shifts for purely Na^+ and Cl^- selective channels are indicated by dashed lines.

(J) Current densities measured at +100 mV for recordings shown in (I).

(K) Relative iodide-over-chloride permeabilities of LRRC8A/C WT and mutants carrying the denoted (or equivalent) mutations in both LRRC8A and LRRC8C subunits. The dotted line reflects the mean WT P_I/P_{Cl}. Boxes show the median and the quartiles of the distribution, and diamonds show outliers that extend beyond the interquartile range multiplied by 1.5.

(L) Current densities measured at +100 mV for the same recordings as in (K). Dotted lines in (J) and (L) reflect the mean WT current.

(M) Time required for the current to reach half of its steady-state level estimated from the same recordings as shown in (K) and (L). *$p < 0.05$; **$p < 0.01$ (Mann-Whitney U test, false discovery rate [FDR] controlled by Benjamini-Hochberg procedure).

Together, these results suggest the presence of two serial selectivity filters in the permeation path, as postulated previously for the CFTR Cl^- channel.[45] Although they may not function totally independently from each other, these two filters work together to determine anion permeability of LRRC8/VRACs. Moreover, the effect of the E6A mutation on activation kinetics (Figure 3M) suggested that NTs also play a role in channel activation.

NTs modulate ionic strength-dependent channel activation

Cytoplasmic LRR domains of LRRC8 channels play important roles in the activation of VRAC by various stimuli,[16,17,24–28,46] but the mechanism by which they couple to a physical gate remains largely enigmatic. Strikingly, deletion of NTs, oxidative modification, or perturbation of NT structure in the LRRC8A

CellPress
OPEN ACCESS

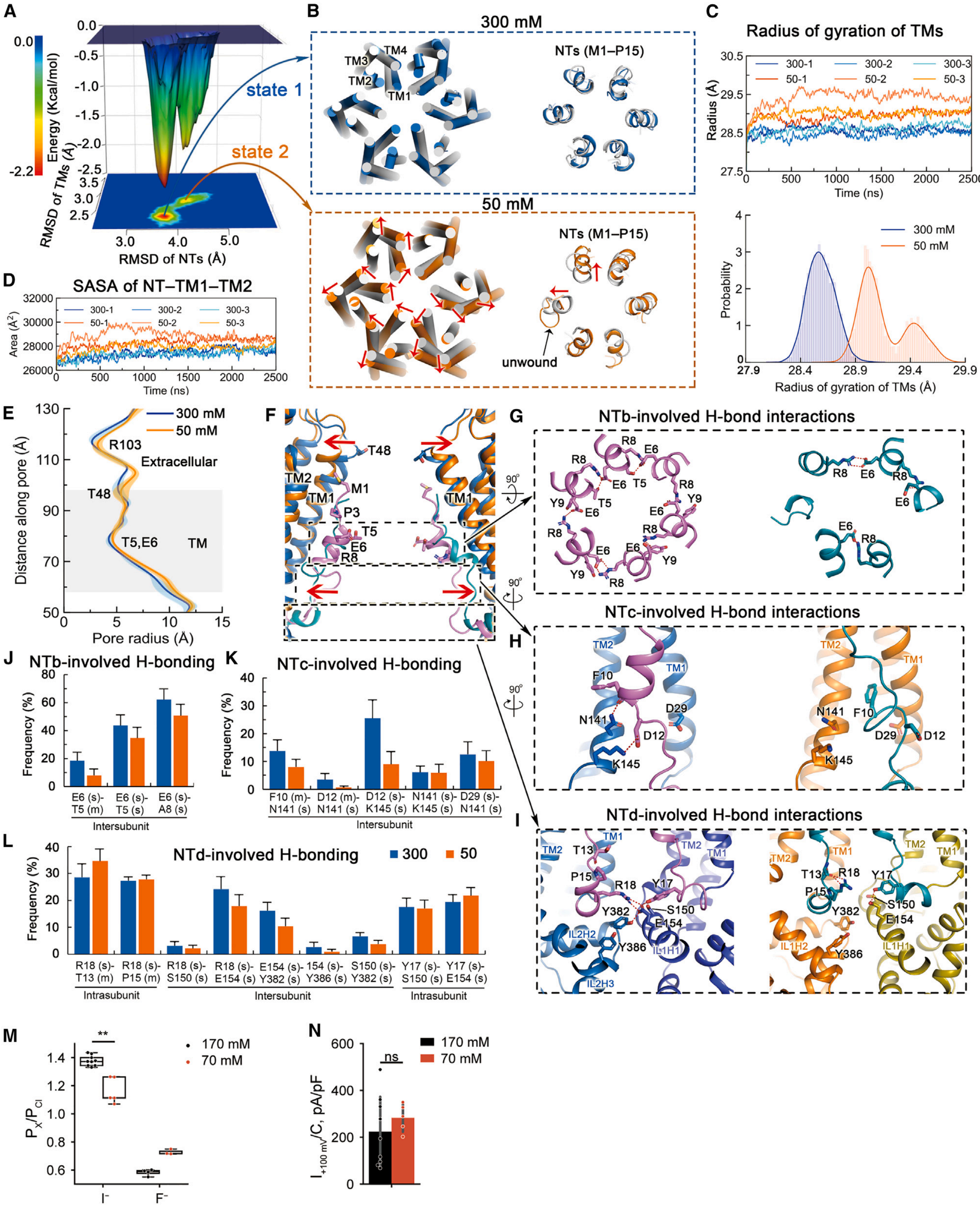

(legend on next page)

subunit affect the activation of LRRC8 channels,[3,20,26] suggesting that NTs are essential for channel activation and might even be a structural component of the gate. We conducted MD simulations for HsLRRC8A at high (300 mM NaCl) and low (50 mM NaCl) ionic strengths to explore whether conformational changes of the NTs and surrounding structures might explain gating of VRAC by low ionic strength[21,22] (Figures 4 and S6–S9). Three independent 2.5-μs simulations were performed for each system that was initiated with the cryo-EM structure of HsLRRC8A. We first calculated the free-energy landscapes as the functions of the RMSDs for NTs ($RMSD_{NTs}$) and for TM domain ($RMSD_{TMs}$) to examine the influence of ionic strength on conformational dynamics of these regions. The landscape revealed different minima at these ionic strengths (Figure 4A). The $RMSD_{NTs}$ and $RMSD_{TMs}$ (Figures 4A, 4B, and S6) displayed larger values at 50 mM NaCl than at 300 mM NaCl. Consistently, root-mean-square fluctuations of the NTs and the TM domain are larger at 50 mM NaCl than at 300 mM NaCl (Figure S7). These results collectively suggested NTs and TM domain become more conformationally flexible upon lowering of ionic strength.

While NTs and TMs show indistinguishable movements in MD trajectories at higher (i.e., 300 mM NaCl) ionic strength, these regions displayed radial dilation at lower ionic strength (i.e., 50 mM NaCl, Figures 4B–4F and S8). Consequently, the radius of gyration of TM domain around the z axis is increased (Figure 4C), and the permeation path is widened (e.g., the radial dilation around R103 residues, Figures 4E and S8), thereby increasing the solvent-accessible surface area (Figure 4D). Moreover, at least one NT was unwound at 50 mM NaCl (Figures 4B, 4F, and S9). These effects greatly reduced the NT-mediated inter- and intra-subunit interactions that stabilize the tightly packed pore domain at high ionic strength (Figures 4G–4L). These interactions include inter-subunit polar interactions between neighboring NTb subsegments (i.e., interactions involving residues T5, E6, and R8 of NTb) (Figures 4G and 4J), intra-subunit H-bond interactions between NTc (i.e., F10 and D12) and TM1 (i.e., D29) and TM2 (i.e., N141 and K145) (Figures 4H and 4K), polar intra-subunit contacts involving NTd (i.e., Y17) and IL1H1 (i.e., S150 and E154), and inter-subunit contacts involving NTd (i.e., R18) in one subunit and TM2 (i.e., E154) in the neighboring subunit (Figures 4I and 4L).

The predicted partial unwinding of the NTs, important parts of the inner selectivity filter, with low ionic strength suggests concomitant changes in ion selectivity. In whole-cell patch-clamp experiments, we tested effects of cytoplasmic ionic strength by replacing 100 mM cesium methanesulfonate in the pipette solution by mannitol and measuring the I^-/Cl^- and F^-/Cl^- permeability ratios of hypotonicity-stimulated native VRAC channels. Indeed, reduction in intracellular ionic strength blunted the channel's ability to discriminate between anions (Figures 4M, 4N, and S5E).

We speculate that differential unwinding of NTs during VRAC activation might lead to different pore structures in the same heteromeric channel, and that these different open states may display different preferences for halide anions and organic substrates. This might explain the finding that highly anion-selective VRAC channels can transport, albeit at a much slower rate, a variety of differently charged or uncharged organic substrates.[7]

Collectively, these calculations agree with the experimentally confirmed importance of LRRC8 NTs in VRAC activation[3,11,20] and with the disturbance of tight LRRC8A assembly by the inclusion of "activating" LRRC8C observed in recently reported cryo-EM structures of LRRC8A/C heteromers.[17,18] They suggest a mechanistic explanation for the activation of VRAC channels by low ionic strength.[21–23]

These results prompt us to propose a model (Figure 5) for NT-mediated activation of LRRC8 channels. It proposes that NTs constitute a gating device[20] that changes its interactions with pore-lining TMs and intracellular loops upon reduced ionic strength. Our findings revealed unique physicochemical and

Figure 4. Ionic strength-dependent conformational changes of NTs and TMs, as predicted by MD simulations and tested by electrophysiology

(A) Free-energy landscapes as the functions of RMSD values of NTs ($RMSD_{NTs}$) and TMs ($RMSD_{TMs}$). The RMSD values of NTs and TMs were generated from the last 1,000-ns trajectories of six independent 2,500-ns simulations at salt concentrations of 300 mM NaCl or 50 mM NaCl.

(B) A representative snapshot of movements of TMs (left) and NTs (right) relative to those of the cryo-EM structure at salt concentrations of 300 mM NaCl (top) or 50 mM NaCl (bottom), respectively. Movements of TMs and NTs in the snapshot are evident for simulations at 50 mM NaCl and are marked by red arrows. An unwound subsegment of NT is annotated by a black arrow.

(C) Radius of gyration of TM domain around the z axis during the course of simulation (top) and probabilities of the radius in the last 1,000-ns simulations (bottom).

(D) Changes in solvent-accessible surface areas (SASAs) of transmembrane permeation path constituted by NTs, TM1s, and TM2s during the course of simulation.

(E) The pore radius at salt concentrations of 300 mM NaCl and 50 mM NaCl, respectively. Radii were calculated from the last 1,000-ns snapshots at 1-ns intervals for each simulation. The transmembrane segment of the pore domain is colored in gray. Data are shown as the mean ± SD of three independent simulations for each system.

(F) Superimposition of pore-lining TMs and NTs of a representative snapshot at 300 mM NaCl to those of a representative snapshot at 50 mM NaCl. The snapshots are identical to those in (B). For clarity, the TMs are colored as in (B), whereas the snapshots of NTs at 300 mM NaCl and 50 mM NaCl are in violet and teal colors, respectively. The radial movements of the snapshot at 50 mM NaCl relative to the snapshot at 300 mM NaCl are marked by red arrows. NTb, NTc, and NTd are highlighted in black dashed boxes.

(G–I) Representative inter- and intra-subunit interactions involving NTb (G), NTc (H), and NTd (I) of the representative snapshots at 300 mM NaCl and 50 mM NaCl, respectively. H-bonds are shown as red dashed lines.

(J–L) Frequencies of typical H-bond interactions involving NTb (J), NTc (K), and NTd (L). m or s in parentheses represent the main-chain atoms or side-chain atoms of a residue that participate in H-bond interactions. Higher values of frequencies represent more stable interactions. Data are shown as the mean ± SD of three independent simulations for each system.

(M) Halide permeability ratios obtained as in Figure 3K from untransfected WT HCT116 cells expressing all LRRC8s at native levels using pipette solutions with low (70 mM) and high (170 mM) ionic strength.

(N) Current densities at +100 mV assessed from the same recordings as in (M). **$p < 0.01$; ns, not significant (Mann-Whitney U test, FDR controlled by Benjamini-Hochberg procedure).

CellPress
OPEN ACCESS

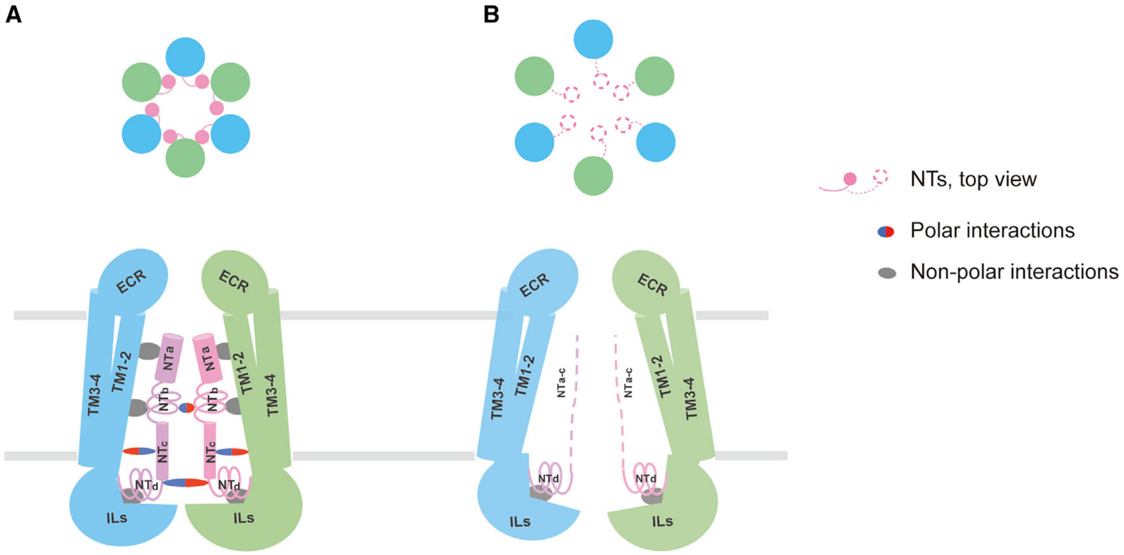

Figure 5. Model of NT-modulated activation of LRRC8 pore domain

Schematic channel model showing cross-section at level of membrane outer leaflet (top) and side view (bottom) in the closed (A) and open (B) state. Two adjacent subunits of the pore domain are shown in the side view. ECR and ILs annotate the extracellular regions and intracellular loops of the pore domain, respectively. NTa through NTc might be conformationally flexible, while the motility of NTd is restricted due to its engagement with other regions of HsLRRC8A by hydrophobic and polar interactions. In the closed state, NTs and pore-surrounding TMs work together to stabilize the tightly packed assembly of the pore domain. Interactions between NTs and pore-surrounding TMs force NTs to restricted conformations and positions (as in A). Our model starts with the ordered NT structures: (1) NTs, and pore-surrounding TMs entrap the transmembrane portion of the pore domain in a packed and partially closed state; (2) the NTs gain conformational flexibility upon reduction of intracellular ionic strength and change their interactions with pore-surrounding TMs and ILs; in particular, NT-mediated inter-subunit interactions are largely reduced; (3) the pore domain expands radially due to the weakened restraint of subunit oligomerization; (4) the transmembrane constriction is disrupted, thereby allowing for the permeation of large molecules and ions. In essence, NTs constitute a gating device[20] that gate channel activation by adjusting their interaction with pore-surrounding TMs and ILs upon reduction of ionic strength. Thus, the pore domain transitions from a tightly packed state into a loosely packed state, and the permeation path switches from the constricted, partially closed conformation to dilated, opened conformation that allows the permeation of both small ions and large molecules.

structural features of NTs that control LRRC8 channels' anion perm-selectivity and activation mechanism that differ from those of other large pore channels.[1,2,5,8,32]

Limitations of the study

Although we have resolved the NTs in the current HsLRRC8A structure, intermediate structures showing NTs prior to complete disordering have not been captured. Structures of NTs of heteromeric LRRC8A/C channels, which were used in functional expression, were not determined. The resolution of LRR domain structures needs to be further improved, and their interactions with intracellular loops and/or NTs remain to be investigated. The computational power and the MD simulations methods are currently insufficient to completely reveal the channel activation process. Restrictions imposed by computation times did not allow to model full activation by low ionic strength. An LRRC8 structure in its activated, open conformation will help us to understand the mechanistic roles of NTs and LRR domains in gating LRRC8 channels.

STAR★METHODS

Detailed methods are provided in the online version of this paper and include the following:

- KEY RESOURCES TABLE
- RESOURCE AVAILABILITY
  - Lead contact
  - Materials availability
  - Data and code availability
- EXPERIMENTAL MODEL AND SUBJECT DETAILS
  - Mammalian cell lines and culture conditions
  - Insect cell lines and culture conditions
- METHOD DETAILS
  - Protein expression and purification
  - Cryo-EM sample preparation and data acquisition
  - Cryo-EM image processing
  - Model building, refinement, and validation
  - Molecular dynamics simulations
  - Steered molecular dynamics simulations
  - Potential-of-mean-force calculations
  - Electrophysiology
- QUANTIFICATION AND STATISTICAL ANALYSIS

SUPPLEMENTAL INFORMATION

Supplemental information can be found online at https://doi.org/10.1016/j.celrep.2023.112926.

ACKNOWLEDGMENTS

We thank the Center of Cryo-Electron Microscopy of ShanghaiTec University for support in collecting cryo-EM data. We also thank the support of ECNU

Multifunctional Platform for Innovation (001) and appreciate Beijing PARATERA Tech Co., Ltd (https://paratera.com/) and National Super Computing Center in Zhengzhou for providing computational resources for this study. This work was funded by the Ministry of Science and Technology of China (2017YFA0504800, 2018ZX09711003-003-003, and 2019ZX097 21001-004-007 to J.L. and 2018YFA0508100 to L.Y. and Q.Z.), ShanghaiTech University (to J.L.), and the National Natural Science Foundation of China (31900895 to L.Y.). It was also supported, in part, by the European Research Council (ERC) Advanced Grant 740537 (VOLSIGNAL) and the Deutsche Forschungsgemeinschaft (DFG) JE164/12-2 and Excellence Strategy—EXC-2049—390688087 grants to T.J.J.

AUTHOR CONTRIBUTIONS

H. Liu, H. Li, and J.W. performed the experiments involving protein purification and cell culture. H. Liu performed cryo-EM grid preparation and data acquisition. H. Liu, and J.L. performed cryo-EM structure determination; M.M.P. performed and M.M.P. and T.J.J. analyzed electrophysiology; L.Y., M.L., and Z.H. performed MD simulations on wild-type HsLRRC8A or the channel mutants. Q.Z. performed steered MD simulations and PMF calculations. All authors contributed to data analysis; J.L., T.J.J., M.M.P., L.Y., Q.Z., and H. Liu wrote the manuscript with input from all authors.

DECLARATION OF INTERESTS

The authors declare no competing interests.

INCLUSION AND DIVERSITY

We support inclusive, diverse, and equitable conduct of research.

Received: December 29, 2022
Revised: June 12, 2023
Accepted: July 18, 2023
Published: August 6, 2023

REFERENCES

1. Jentsch, T.J. (2016). VRACs and other ion channels and transporters in the regulation of cell volume and beyond. Nat. Rev. Mol. Cell Biol. *17*, 293–307. https://doi.org/10.1038/nrm.2016.29.
2. Strange, K., Yamada, T., and Denton, J.S. (2019). A 30-year journey from volume-regulated anion currents to molecular structure of the LRRC8 channel. J. Gen. Physiol. *151*, 100–117. https://doi.org/10.1085/jgp.201812138.
3. Voss, F.K., Ullrich, F., Münch, J., Lazarow, K., Lutter, D., Mah, N., Andrade-Navarro, M.A., von Kries, J.P., Stauber, T., and Jentsch, T.J. (2014). Identification of LRRC8 Heteromers as an Essential Component of the Volume-Regulated Anion Channel VRAC. Science *344*, 634–638. https://doi.org/10.1126/science.1252826.
4. Qiu, Z., Dubin, A.E., Mathur, J., Tu, B., Reddy, K., Miraglia, L.J., Reinhardt, J., Orth, A.P., and Patapoutian, A. (2014). SWELL1, a Plasma Membrane Protein, Is an Essential Component of Volume-Regulated Anion Channel. Cell *157*, 447–458. https://doi.org/10.1016/j.cell.2014.03.024.
5. Abascal, F., and Zardoya, R. (2012). LRRC8 proteins share a common ancestor with pannexins, and may form hexameric channels involved in cell-cell communication. Bioessays *34*, 551–560. https://doi.org/10.1002/bies.201100173.
6. Planells-Cases, R., Lutter, D., Guyader, C., Gerhards, N.M., Ullrich, F., Elger, D.A., Kucukosmanoglu, A., Xu, G., Voss, F.K., Reincke, S.M., et al. (2015). Subunit composition of VRAC channels determines substrate specificity and cellular resistance to Pt-based anti-cancer drugs. EMBO J. *34*, 2993–3008. https://doi.org/10.15252/embj.201592409.
7. Lutter, D., Ullrich, F., Lueck, J.C., Kempa, S., and Jentsch, T.J. (2017). Selective transport of neurotransmitters and modulators by distinct volume-regulated LRRC8 anion channels. J. Cell Sci. *130*, 1122–1133. https://doi.org/10.1242/jcs.196253.
8. Nilius, B., Eggermont, J., Voets, T., Buyse, G., Manolopoulos, V., and Droogmans, G. (1997). Properties of volume-regulated anion channels in mammalian cells. Prog. Biophys. Mol. Biol. *68*, 69–119. https://doi.org/10.1016/S0079-6107(97)00021-7.
9. Pedersen, S.F., Klausen, T.K., and Nilius, B. (2015). The identification of a volume-regulated anion channel: an amazing Odyssey. Acta Physiol. *213*, 868–881. https://doi.org/10.1111/apha.12450.
10. Ullrich, F., Reincke, S.M., Voss, F.K., Stauber, T., and Jentsch, T.J. (2016). Inactivation and Anion Selectivity of Volume-regulated Anion Channels (VRACs) Depend on C-terminal Residues of the First Extracellular Loop. J. Biol. Chem. *291*, 17040–17048. https://doi.org/10.1074/jbc.M116.739342.
11. Kefauver, J.M., Saotome, K., Dubin, A.E., Pallesen, J., Cottrell, C.A., Cahalan, S.M., Qiu, Z., Hong, G., Crowley, C.S., Whitwam, T., et al. (2018). Structure of the human volume regulated anion channel. Elife *7*, e38461. https://doi.org/10.7554/eLife.38461.
12. Kasuya, G., Nakane, T., Yokoyama, T., Jia, Y., Inoue, M., Watanabe, K., Nakamura, R., Nishizawa, T., Kusakizako, T., Tsutsumi, A., et al. (2018). Cryo-EM structures of the human volume-regulated anion channel LRRC8. Nat. Struct. Mol. Biol. *25*, 797–804. https://doi.org/10.1038/s41594-018-0109-6.
13. Deneka, D., Sawicka, M., Lam, A.K.M., Paulino, C., and Dutzler, R. (2018). Structure of a volume-regulated anion channel of the LRRC8 family. Nature *558*, 254–259. https://doi.org/10.1038/s41586-018-0134-y.
14. Kern, D.M., Oh, S., Hite, R.K., and Brohawn, S.G. (2019). Cryo-EM structures of the DCPIB-inhibited volume-regulated anion channel LRRC8A in lipid nanodiscs. Elife *8*, e42636. https://doi.org/10.7554/eLife.42636.
15. Nakamura, R., Numata, T., Kasuya, G., Yokoyama, T., Nishizawa, T., Kusakizako, T., Kato, T., Hagino, T., Dohmae, N., Inoue, M., et al. (2020). Cryo-EM structure of the volume-regulated anion channel LRRC8D isoform identifies features important for substrate permeation. Commun. Biol. *3*, 240. https://doi.org/10.1038/s42003-020-0951-z.
16. Deneka, D., Rutz, S., Hutter, C.A.J., Seeger, M.A., Sawicka, M., and Dutzler, R. (2021). Allosteric modulation of LRRC8 channels by targeting their cytoplasmic domains. Nat. Commun. *12*, 5435. https://doi.org/10.1038/s41467-021-25742-w.
17. Rutz, S., Deneka, D., Dittmann, A., Sawicka, M., and Dutzler, R. (2023). Structure of a volume-regulated heteromeric LRRC8A/C channel. Nat. Struct. Mol. Biol. *30*, 52–61. https://doi.org/10.1038/s41594-022-00899-0.
18. Kern, D.M., Bleier, J., Mukherjee, S., Hill, J.M., Kossiakoff, A.A., Isacoff, E.Y., and Brohawn, S.G. (2023). Structural basis for assembly and lipid-mediated gating of LRRC8A:C volume-regulated anion channels. Nat. Struct. Mol. Biol. *30*, 841–852. https://doi.org/10.1038/s41594-023-00944-6.
19. Yamada, T., and Strange, K. (2018). Intracellular and extracellular loops of LRRC8 are essential for volume-regulated anion channel function. J. Gen. Physiol. *150*, 1003–1015. https://doi.org/10.1085/jgp.201812016.
20. Zhou, P., Polovitskaya, M.M., and Jentsch, T.J. (2018). LRRC8 N termini influence pore properties and gating of volume-regulated anion channels (VRACs). J. Biol. Chem. *293*, 13440–13451. https://doi.org/10.1074/jbc.RA118.002853.
21. Cannon, C.L., Basavappa, S., and Strange, K. (1998). Intracellular ionic strength regulates the volume sensitivity of a swelling-activated anion channel. Am. J. Physiol. *275*, C416–C422. https://doi.org/10.1152/ajpcell.1998.275.2.C416.
22. Voets, T., Droogmans, G., Raskin, G., Eggermont, J., and Nilius, B. (1999). Reduced intracellular ionic strength as the initial trigger for activation of endothelial volume-regulated anion channels. Proc. Natl. Acad. Sci. USA *96*, 5298–5303. https://doi.org/10.1073/pnas.96.9.5298.

CellPress
OPEN ACCESS

23. Syeda, R., Qiu, Z., Dubin, A.E., Murthy, S.E., Florendo, M.N., Mason, D.E., Mathur, J., Cahalan, S.M., Peters, E.C., Montal, M., and Patapoutian, A. (2016). LRRC8 Proteins Form Volume-Regulated Anion Channels that Sense Ionic Strength. Cell *164*, 499–511. https://doi.org/10.1016/j.cell.2015.12.031.

24. König, B., Hao, Y., Schwartz, S., Plested, A.J., and Stauber, T. (2019). A FRET sensor of C-terminal movement reveals VRAC activation by plasma membrane DAG signaling rather than ionic strength. Elife *8*, e45421. https://doi.org/10.7554/elife.45421.

25. Bertelli, S., Remigante, A., Zuccolini, P., Barbieri, R., Ferrera, L., Picco, C., Gavazzo, P., and Pusch, M. (2021). Mechanisms of Activation of LRRC8 Volume Regulated Anion Channels. Cell. Physiol. Biochem. *55*, 41–56. https://doi.org/10.33594/000000329.

26. Bertelli, S., Zuccolini, P., Gavazzo, P., and Pusch, M. (2022). Molecular determinants underlying volume-regulated anion channel subunit-dependent oxidation sensitivity. J. Physiol. *600*, 3965–3982. https://doi.org/10.1113/JP283321.

27. Sawicka, M., and Dutzler, R. (2022). Regulators of cell volume: The structural and functional properties of anion channels of the LRRC8 family. Curr. Opin. Struct. Biol. *74*, 102382. https://doi.org/10.1016/j.sbi.2022.102382.

28. Kasuya, G., and Nureki, O. (2022). Recent Advances in the Structural Biology of the Volume-Regulated Anion Channel LRRC8. Front. Pharmacol. *13*, 896532. https://doi.org/10.3389/fphar.2022.896532.

29. Takahashi, H., Yamada, T., Denton, J.S., Strange, K., and Karakas, E. (2023). Cryo-EM structures of an LRRC8 chimera with native functional properties reveal heptameric assembly. Elife *12*, e82431. https://doi.org/10.7554/eLife.82431.

30. Huang, J., and MacKerell, A.D., Jr. (2013). CHARMM36 all-atom additive protein force field: validation based on comparison to NMR data. J. Comput. Chem. *34*, 2135–2145. https://doi.org/10.1002/jcc.23354.

31. Smart, O.S., Goodfellow, J.M., and Wallace, B.A. (1993). The pore dimensions of gramicidin A. Biophys. J. *65*, 2455–2460. https://doi.org/10.1016/S0006-3495(93)81293-1.

32. Syrjanen, J., Michalski, K., Kawate, T., and Furukawa, H. (2021). On the molecular nature of large-pore channels. J. Mol. Biol. *433*, 166994. https://doi.org/10.1016/j.jmb.2021.166994.

33. Kuzuya, M., Hirano, H., Hayashida, K., Watanabe, M., Kobayashi, K., Terada, T., Mahmood, M.I., Tama, F., Tani, K., Fujiyoshi, Y., and Oshima, A. (2022). Structures of human pannexin-1 in nanodiscs reveal gating mediated by dynamic movement of the N terminus and phospholipids. Sci. Signal. *15*, eabg6941. https://doi.org/10.1126/scisignal.abg6941.

34. Myers, J.B., Haddad, B.G., O'Neill, S.E., Chorev, D.S., Yoshioka, C.C., Robinson, C.V., Zuckerman, D.M., and Reichow, S.L. (2018). Structure of native lens connexin 46/50 intercellular channels by cryo-EM. Nature *564*, 372–377. https://doi.org/10.1038/s41586-018-0786-7.

35. Maeda, S., Nakagawa, S., Suga, M., Yamashita, E., Oshima, A., Fujiyoshi, Y., and Tsukihara, T. (2009). Structure of the connexin 26 gap junction channel at 3.5 Å resolution. Nature *458*, 597–602. https://doi.org/10.1038/nature07869.

36. Oshima, A., Tani, K., and Fujiyoshi, Y. (2016). Atomic structure of the innexin-6 gap junction channel determined by cryo-EM. Nat. Commun. *7*, 13681. https://doi.org/10.1038/ncomms13681.

37. Ruan, Z., Orozco, I.J., Du, J., and Lü, W. (2020). Structures of human pannexin 1 reveal ion pathways and mechanism of gating. Nature *584*, 646–651. https://doi.org/10.1038/s41586-020-2357-y.

38. Droogmans, G., Maertens, C., Prenen, J., and Nilius, B. (1999). Sulphonic acid derivatives as probes of pore properties of volume-regulated anion channels in endothelial cells. Br. J. Pharmacol. *128*, 35–40. https://doi.org/10.1038/sj.bjp.0702770.

39. Ternovsky, V.I., Okada, Y., and Sabirov, R.Z. (2004). Sizing the pore of the volume-sensitive anion channel by differential polymer partitioning. FEBS Lett. *576*, 433–436. https://doi.org/10.1016/j.febslet.2004.09.051.

40. Bogunia, M., and Makowski, M. (2020). Influence of Ionic Strength on Hydrophobic Interactions in Water: Dependence on Solute Size and Shape. J. Phys. Chem. B *124*, 10326–10336. https://doi.org/10.1021/acs.jpcb.0c06399.

41. Yamada, T., Figueroa, E.E., Denton, J.S., and Strange, K. (2021). LRRC8A homohexameric channels poorly recapitulate VRAC regulation and pharmacology. Am. J. Physiol. Cell Physiol. *320*, C293–C303. https://doi.org/10.1152/ajpcell.00454.2020.

42. Cheng, M.H., and Coalson, R.D. (2012). Molecular Dynamics Investigation of Cl− and Water Transport through a Eukaryotic CLC Transporter. Biophys. J. *102*, 1363–1371. https://doi.org/10.1016/j.bpj.2012.01.056.

43. Yue, Z., Wang, Z., and Voth, G.A. (2022). Ion permeation, selectivity, and electronic polarization in fluoride channels. Biophys. J. *121*, 1336–1347. https://doi.org/10.1016/j.bpj.2022.02.019.

44. Dutzler, R., Campbell, E.B., Cadene, M., Chait, B.T., and MacKinnon, R. (2002). X-ray structure of a ClC chloride channel at 3.0 A reveals the molecular basis of anion selectivity. Nature *415*, 287–294. https://doi.org/10.1038/415287a.

45. Linsdell, P. (2016). Anion conductance selectivity mechanism of the CFTR chloride channel. Biochim. Biophys. Acta *1858*, 740–747. https://doi.org/10.1016/j.bbamem.2016.01.009.

46. Gaitán-Peñas, H., Gradogna, A., Laparra-Cuervo, L., Solsona, C., Fernández-Dueñas, V., Barrallo-Gimeno, A., Ciruela, F., Lakadamyali, M., Pusch, M., and Estévez, R. (2016). Investigation of LRRC8-Mediated Volume-Regulated Anion Currents in Xenopus Oocytes. Biophys. J. *111*, 1429–1443. https://doi.org/10.1016/j.bpj.2016.08.030.

47. Emsley, P., Lohkamp, B., Scott, W.G., and Cowtan, K. (2010). Features and development of Coot. Acta Crystallogr. D Biol. Crystallogr. *66*, 486–501. https://doi.org/10.1107/s0907444910007493.

48. Adams, P.D., Afonine, P.V., Bunkóczi, G., Chen, V.B., Davis, I.W., Echols, N., Headd, J.J., Hung, L.W., Kapral, G.J., Grosse-Kunstleve, R.W., et al. (2010). PHENIX: a comprehensive Python-based system for macromolecular structure solution. Acta Crystallogr. D Biol. Crystallogr. *66*, 213–221. https://doi.org/10.1107/s0907444909052925.

49. Scheres, S.H.W. (2012). RELION: implementation of a Bayesian approach to cryo-EM structure determination. J. Struct. Biol. *180*, 519–530. https://doi.org/10.1016/j.jsb.2012.09.006.

50. Punjani, A., Rubinstein, J.L., Fleet, D.J., and Brubaker, M.A. (2017). cryoSPARC: algorithms for rapid unsupervised cryo-EM structure determination. Nat. Methods *14*, 290–296. https://doi.org/10.1038/nmeth.4169.

51. Mastronarde, D.N. (2005). Automated electron microscope tomography using robust prediction of specimen movements. J. Struct. Biol. *152*, 36–51. https://doi.org/10.1016/j.jsb.2005.07.007.

52. Pettersen, E.F., Goddard, T.D., Huang, C.C., Couch, G.S., Greenblatt, D.M., Meng, E.C., and Ferrin, T.E. (2004). UCSF Chimera–a visualization system for exploratory research and analysis. J. Comput. Chem. *25*, 1605–1612. https://doi.org/10.1002/jcc.20084.

53. Goddard, T.D., Huang, C.C., Meng, E.C., Pettersen, E.F., Couch, G.S., Morris, J.H., and Ferrin, T.E. (2018). UCSF ChimeraX: Meeting modern challenges in visualization and analysis. Protein Sci. *27*, 14–25. https://doi.org/10.1002/pro.3235.

54. Páll, S., Zhmurov, A., Bauer, P., Abraham, M., Lundborg, M., Gray, A., Hess, B., and Lindahl, E. (2020). Heterogeneous parallelization and acceleration of molecular dynamics simulations in GROMACS. J. Chem. Phys. *153*, 134110. https://doi.org/10.1063/5.0018516.

55. Ponimaskin, E.G., Profirovic, J., Vaiskunaite, R., Richter, D.W., and Voyno-Yasenetskaya, T.A. (2002). 5-Hydroxytryptamine 4(a) receptor is coupled to the Galpha subunit of heterotrimeric G13 protein. J. Biol. Chem. *277*, 20812–20819. https://doi.org/10.1074/jbc.M112216200.

56. Reeves, P.J., Callewaert, N., Contreras, R., and Khorana, H.G. (2002). Structure and function in rhodopsin: high-level expression of rhodopsin with restricted and homogeneous N-glycosylation by a tetracycline-inducible N-acetylglucosaminyltransferase I-negative HEK293S stable

mammalian cell line. Proc. Natl. Acad. Sci. USA *99*, 13419–13424. https://doi.org/10.1073/pnas.212519299.

57. Goehring, A., Lee, C.H., Wang, K.H., Michel, J.C., Claxton, D.P., Baconguis, I., Althoff, T., Fischer, S., Garcia, K.C., and Gouaux, E. (2014). Screening and large-scale expression of membrane proteins in mammalian cells for structural studies. Nat. Protoc. *9*, 2574–2585. https://doi.org/10.1038/nprot.2014.173.

58. Zheng, S.Q., Palovcak, E., Armache, J.P., Verba, K.A., Cheng, Y., and Agard, D.A. (2017). MotionCor2: anisotropic correction of beam-induced motion for improved cryo-electron microscopy. Nat. Methods *14*, 331–332. https://doi.org/10.1038/nmeth.4193.

59. Zhang, K. (2016). Gctf: Real-time CTF determination and correction. J. Struct. Biol. *193*, 1–12. https://doi.org/10.1016/j.jsb.2015.11.003.

60. Scheres, S.H.W., and Chen, S. (2012). Prevention of overfitting in cryo-EM structure determination. Nat. Methods *9*, 853–854. https://doi.org/10.1038/nmeth.2115.

61. Chen, S., McMullan, G., Faruqi, A.R., Murshudov, G.N., Short, J.M., Scheres, S.H.W., and Henderson, R. (2013). High-resolution noise substitution to measure overfitting and validate resolution in 3D structure determination by single particle electron cryomicroscopy. Ultramicroscopy *135*, 24–35. https://doi.org/10.1016/j.ultramic.2013.06.004.

62. Rosenthal, P.B., and Henderson, R. (2003). Optimal determination of particle orientation, absolute hand, and contrast loss in single-particle electron cryomicroscopy. J. Mol. Biol. *333*, 721–745. https://doi.org/10.1016/j.jmb.2003.07.013.

63. Miyamoto, S., and Kollman, P.A. (1992). Settle: An analytical version of the SHAKE and RATTLE algorithm for rigid water models. J. Comput. Chem. *13*, 952–962. https://doi.org/10.1002/jcc.540130805.

64. Hess, B., Bekker, H., Berendsen, H.J.C., and Fraaije, J.G.E.M. (1997). LINCS: A linear constraint solver for molecular simulations. J. Comput. Chem. *18*, 1463–1472. https://doi.org/10.1002/(SICI)1096-987X(199709)18:12<1463::AID-JCC4>3.0.CO;2-H.

65. Essmann, U., Perera, L., Berkowitz, M.L., Darden, T., Lee, H., and Pedersen, L.G. (1995). A smooth particle mesh Ewald method. J. Chem. Phys. *103*, 8577–8593. https://doi.org/10.1063/1.470117.

66. Bussi, G., Donadio, D., and Parrinello, M. (2007). Canonical sampling through velocity rescaling. J. Chem. Phys. *126*, 014101. https://doi.org/10.1063/1.2408420.

67. Parrinello, M., and Rahman, A. (1981). Polymorphic transitions in single crystals: A new molecular dynamics method. J. Appl. Phys. *52*, 7182–7190. https://doi.org/10.1063/1.328693.

68. Izrailev, S., Crofts, A.R., Berry, E.A., and Schulten, K. (1999). Steered molecular dynamics simulation of the Rieske subunit motion in the cytochrome bc(1) complex. Biophys. J. *77*, 1753–1768. https://doi.org/10.1016/s0006-3495(99)77022-0.

69. Torrie, G.M., and Valleau, J.P. (1977). Nonphysical sampling distributions in Monte Carlo free-energy estimation: Umbrella sampling. J. Comput. Phys. *23*, 187–199. https://doi.org/10.1016/0021-9991(77)90121-8.

70. Kumar, S., Rosenberg, J.M., Bouzida, D., Swendsen, R.H., and Kollman, P.A. (1992). THE weighted histogram analysis method for free-energy calculations on biomolecules. I. The method. J. Comput. Chem. *13*, 1011–1021. https://doi.org/10.1002/jcc.540130812.

71. Virtanen, P., Gommers, R., Oliphant, T.E., Haberland, M., Reddy, T., Cournapeau, D., Burovski, E., Peterson, P., Weckesser, W., Bright, J., et al. (2020). SciPy 1.0: fundamental algorithms for scientific computing in Python. Nat. Methods *17*, 261–272. https://doi.org/10.1038/s41592-019-0686-2.

CellPress
OPEN ACCESS

STAR★METHODS

KEY RESOURCES TABLE

REAGENT or RESOURCE	SOURCE	IDENTIFIER
Antibodies		
Anti-His (C-term)-HRP antibody	Abmart	Cat# M20020S
Anti-Flag-HRP antibody	Abmart	Cat# PA9020S
Bacterial and virus strains		
E. coli BL21 (DE3)	Thermo Fisher Scientific	Cat# EC0114
E.coli DH5α	Thermo Fisher Scientific	Cat# 18265017
E.coli Rosetta-gami B(DE3)	Merck Millipore	Cat# 71136
E.coli DH10Bac	Thermo Fisher Scientific	Cat# 10361012
Chemicals, peptides, and recombinant proteins		
Dodecyl -D-maltoside (DDM)	Anatrace	Cat# D310LA
Cholesteryl hemisuccinate (CHS)	Anatrace	Cat# CH210
GDN	Anatrace	Cat# GDN101
DYKDDDDK G1 Affinity Resin	GenScript	Cat# L00432
DYKDDDDK peptide	Genscript	Cat# RP10586-1
Aprotinin	Merck Millipore	Cat# A1250000
Leupeptin	Merck Millipore	Cat# L5793
Pepstatin A	Merck Millipore	Cat# 77170
L-glutamine	Thermo Fisher Scientific	Cat# 25030081
Sodium pyruvate	Thermo Fisher Scientific	Cat# 11360070
GlutaMax	Thermo Fisher Scientific	Cat# 35050061
Penicillin–streptomycin	Thermo Fisher Scientific	Cat# 15140122
Fetal bovine serum (FBS)	Thermo Fisher Scientific	Cat# 10099141
McCoy's 5A Medium	Pan Biotech	Cat# P04-05500
FBS Good	Pan Biotech	Cat# P40-37500
penicillin/streptomycin	Pan Biotech	Cat# P06-07100
Lipofectamine 2000	Thermo Fisher Scientific	Cat# 11668019
Deposited data		
Coordinates of human LRRC8A	This study	PDB: 7XZH
Cryo-EM map of human LRRA8A	This study	EMDB: EMD-33527
Experimental models: Cell lines		
Human: HEK293S GnTI^{-} cells	ATCC	ATCC CRL-3022; RRID: CVCL_A785
Spodoptera frugiperda in Sf-900 II SFM medium	Thermo Fisher Scientific	Cat# 11496015
HCT116 *LRRC8*$^{-/-}$	Voss et al.[3]	N/A
Oligonucleotides		
pEG BacMam-LRRC8A-His-Flag Forward: GCGCG CGGAATTCGCCACCATGATTCCGGTGACAGAGC	This study	N/A
pEG BacMam-LRRC8A -His-Flag Reverse: GGGCT GACAAGGAGCAGGCCTCTAGAGCCTGCAG	This study	N/A
LRRC8A-R103A: CGTAGTTGTACTGGTGCGCGTCCAGGTCATACTTG ATCAAGTATGACCTGGACGCGCACCAGTACAACTACG	This study	N/A
LRRC8C-L105A: TTATAAAGCTGTACTGCTGAGCGTCCAAATCTGTC TTCAGGCGCCTGAAGACAGATTTGGACGCTCAGC AGTACAGCTTTATAA	This study	N/A

(*Continued on next page*)

Continued		
REAGENT or RESOURCE	SOURCE	IDENTIFIER
Recombinant DNA		
pEG BacMam	Eric Gouaux's Lab	RRID: Addgene _160451
pEG BacMam-LRRC8A-His-Flag	This study	N/A
pcDNA3.1_LRRC8A	Voss et al.[3]	N/A
pEGFP_N1_LRRC8C	Voss et al.[3]	N/A
pcDNA3.1_LRRC8A-E6A	Zhou et al.[20]	N/A
pEGFP_N1_LRRC8C-E6A	Zhou et al.[20]	N/A
pcDNA3.1_LRRC8A-R8A	Zhou et al.[20]	N/A
pEGFP_N1_LRRC8C-R8A	Zhou et al.[20]	N/A
pcDNA3.1_LRRC8A-R103A	This study	N/A
pEGFP_N1_LRRC8C-L105A	This study	N/A
pcDNA3.1_LRRC8A-E6A,R103A	This study	N/A
pEGFP_N1_LRRC8C-E6A,L105A	This study	N/A
pcDNA3.1_LRRC8A-R8A,R103A	This study	N/A
pEGFP_N1_LRRC8C-R8A,L105A	This study	N/A
Software and algorithms		
PyMOL	Schrödinger LLC	RRID: SCR_000305
COOT	Emsley et al.[47]	RRID: SCR_014222
PHENIX	Adams et al.[48]	RRID: SCR_014224
RELION 3.0	Scheres et al.[49]	RRID: SCR_016274
cryoSPARC 2.15	Punjani et al.[50]	RRID: SCR_016501
Prism Software version 8	GraphPad Software	RRID: SCR_002798
SerialEM	Mastronarde et al.[51]	RRID: SCR_017293
UCSF Chimera	Pettersen et al.[52]	RRID: SCR_002959
UCSF ChimeraX	Goddard et al.[53]	RRID: SCR_015872
HOLE	Smart et al.[31]	https://www.holeprogram.org/
Gromacs v.2020.2	Páll et al.[54]	RRID: SCR_014565
Other		
Quantifoil R1.2/1.3 Cu, 300 mesh	Quantifoil	Cat# 4230G-CF
KIMBLE Dounce tissue grinder	Merck Millipore	Cat# D9063
Superose 6 Increase 10/300 column	GE Healthcare	Cat# 29091596
Superdex 200 Increase 10/300 GL column	GE Healthcare	Cat# 28990944

RESOURCE AVAILABILITY

Lead contact

Further information and requests for reagents and source data should be directed to and will be fulfilled by the lead contact, Jun Liao (liaojun@shanghaitech.edu.cn).

Materials availability

Plasmids generated in this study are available from the lead contact, but a payment and/or a completed Materials Transfer Agreement may be required if a potential for commercial application exists.

Data and code availability

- Structures and coordinates have been deposited in the Protein DataBank with identification number PDB: 7XZH. Cryo-EM maps have been deposited to the Electron Microscopy DataBank (EMDB) under accession ID EMDB: EMD-33527.
- This paper does not report original code.
- Any additional information required to reanalyze the data reported in this work is available from the lead contact upon request.

EXPERIMENTAL MODEL AND SUBJECT DETAILS

Mammalian cell lines and culture conditions

For structural studies, HsLRRC8A were produced in HEK293S GnTI⁻ cells (ATCC no. CRL-3022) cultured in Freestyle 293 medium (Thermo Fisher Scientific) supplemented with 2% heat-inactivated FBS (Thermo Fisher Scientific), 2 mM L-glutamine (Thermo Fisher Scientific), 1 mM sodium pyruvate (Thermo Fisher Scientific), and 100 U ml−1 penicillin–streptomycin (Thermo Fisher Scientific) at 37°C and 5% CO_2.

The electrophysiological recordings were conducted in HCT116 *LRRC8*$^{-/-}$ cells[3] and WT HCT116 cells cultured in McCoy's 5A medium (PAN Biotech) supplemented with 10% FBS (PAN Biotech) and 1% penicillin/streptomycin (PAN Biotech) at 37°C and 5% CO_2.

Insect cell lines and culture conditions

To overexpress HsLRRC8A for structural studies, recombinant baculovirus for HsLRRC8A was produced in *Spodoptera frugiperda* (Sf9) cells maintained in Sf-900 II SFM medium (Thermo Fisher Scientific) with 10% FBS (Thermo Fisher Scientific) and GlutaMAX (Thermo Fisher Scientific).

METHOD DETAILS

Protein expression and purification

The gene encoding HsLRRC8A (NCBI: txid9606) was synthesized by Genewiz (https://www.genewiz.com.cn/) and was then cloned into pEG BacMam vector with a C-terminal FLAG tag connected by a Rhinovirus 3C protease-cleavable linker. Recombinant baculovirus of HsLRRC8A was generated using the Bac-to-Bac system following a standard protocol.[55] Then HEK293S GnTI$^-$ cells[56] at a density of 2.5–3.0×10^6 cells/mL were infected with baculovirus at a MOI of 5. HEK293S GnTI$^-$ cells were grown in Freestyle 293 medium (Thermo Fisher Scientific) supplemented with 2% heat-inactivated FBS (Thermo Fisher Scientific), 2 mM L-glutamine (Thermo Fisher Scientific), 1 mM sodium pyruvate (Thermo Fisher Scientific), and 100 U ml^{-1} penicillin–streptomycin (Thermo Fisher Scientific) at 37°C and 5% CO_2. Cells were harvested 3 days post-infection[57] and collected by centrifugation. The cell pellets were flash-frozen in liquid nitrogen and stored at −80°C for further use.

The entire purification process was carried out at 4°C. Cell pellets were resuspended in lysis buffer containing 50 mM HEPES pH 8.0, 350 mM NaCl and 1.0% (w/v) n-Dodecyl-B-D-Maltoside (DDM, Anatrace), supplemented with 0.1% (w/v) cholesteryl hemisuccinate (CHS), 50 μg mL^{-1} DNase and RNase A (Sinopharm) and protease inhibitor cocktail (Sigma). The mixture was incubated for 2.5 h under gentle agitation, followed by centrifugation at 20,000 g for 35 min to remove debris. The supernatant containing the solubilized protein was incubated with anti-flag resin (GenScript) for 2 h under gentle agitation. The resin was subsequently washed with 20 column volume of buffer containing 50 mM HEPES pH 8.0, 350 mM NaCl, 0.1% (w/v) glyco-diosgenin (GDN) and 0.01% (w/v) CHS supplemented with protease inhibitor cocktail. The protein was then eluted by buffer containing 50 mM HEPES pH 8.0, 350 mM NaCl, 0.06% (w/v) GDN and 0.006% (w/v) CHS supplemented with 400 μg/mL Flag (DYKDDDDK) peptide (GenScript) and protease inhibitor cocktail. The sample was concentrated using a centrifugal filter (Amicon, 100 kDa molecular weight cut-off), filtered (Millipore, 0.22 μm) and separated on a Superose 6 Increase, 10/300 GL column (GE Healthcare) equilibrated with 20 mM HEPES pH 7.4, 300 mM NaCl, 0.06% (w/v) digitonin and 0.006% (w/v) CHS. Selected peak fractions containing the protein were pooled and concentrated to 16 mg/mL using a centrifugal filter (Ambion, 100 kDa molecular weight cut-off) for immediate cryo-EM grid preparation.

Cryo-EM sample preparation and data acquisition

3 μL samples of the purified protein at a concentration of 16 mg/mL were applied to glow-discharged holey carbon grids (Quantifoil R1.2/1.3 Au 300 mesh). Excess liquid was removed in Vitrobot Mark IV (Thermo Fisher Scientific) under 4°C and 100% humidity conditions by blotting grids for 3.5 s (with a blotting force of 1). Sample loading and blotting was repeated twice. Grids were subsequently flash-frozen in liquid ethane. LRRC8A were imaged in a 300 kV Titan Krios (FEI) with a 70 μm objective aperture. All data were collected using a post-column quantum energy filter (Gatan) with a 20 eV slit and a K2 Summit direct detector (Gatan) operating in super-resolution mode. Dose-fractionated micrographs were recorded in an automated manner using SerialEM[51] with a defocus range of −1.0 to −2.2 μm. Dataset were recorded at a pixel size of 1.04 Å per pixel (0.52 Å per pixel in super-resolution) with a total dose of 60 e/$Å^2$ (40 individual frames).

Cryo-EM image processing

A total of 1950 dose-fractioned images were used for correction of beam-induced movement using a dose-weighting scheme in MotionCor2[58] and their contrast transfer function parameters were estimated by GCTF.[59] With a box size of 300 pixels, 4000 particles were manually picked from 100 images and subsequently subjected to an initial reference-free 2D classification. From this set, five distinctive 2D class averages were selected and used as templates for automated particle picking, which generated a starting dataset of 378,119 particles using Relion3.0.[49] After four rounds of 2D classification, 155,965 particles were retained and imported into CryoSPARC v2.15[50] for ab-initio reconstruction. Three initial models were used as 3D volume templates for two rounds heterogeneous refinement with all selected particles. 75,634 particles were converged into one class that has shown the same conformation

with a clear 3-fold symmetry in the cytoplasmic LRR domain and a 6-fold symmetry in the transmembrane pore domain. Then, this particle set was used to perform homogeneous refinement followed by non-uniform refinement with C1 symmetry. This yielded a model at resolution of 4.36 Å, which was input into Relion3.0[49] for two rounds of 3D classification. The best class contained 31,640 particles and was further refined in CryoSPARC v2.15[50] by homogeneous refinement followed by non-uniform refinement with C1 symmetry, which yielded a model at 3.14 Å resolution. The further non-uniform refinement with C3 symmetry yielded a model with an overall resolution of 2.78 Å. In all cases resolution was estimated in the presence of a soft solvent mask and based on the gold standard Fourier shell correlation (FSC) 0.143 criterion.[60–62] Local resolution was estimated in cryoSPARC v2.15 using default parameters. Unless indicated otherwise, the maps shown in figures were sharpened with B factors estimated in the nonuniform refinement and low-pass filtered at their resolution.

Model building, refinement, and validation

The model of HsLRRC8A was built in Coot.[47] The cryo-EM density generated with C3 symmetry was of high quality and enabled the unambiguous assignment of residues. The pore domain of HsLRRC8A, including N-terminal extensions, were built into the electron density using mouse LRRC8A (PDB 6G9O) structure as a guide. The LRR domain was initially generated by creating a homology model based on the LRR domain (PDB 6FNW) of mouse LRRC8A. Refinements were performed using phenix.real_space_refine in PHENIX[48] with secondary structure and geometry restraints, in combination with manual building. The statistics of model validation were shown in Table S1. UCSF Chimera,[52] Chimera X,[53] PyMOL (https://pymol.org), and HOLE[31] were used to prepare the cryo-EM structural figures in the paper.

Molecular dynamics simulations

We performed molecular dynamics (MD) simulations for the pore domain (M1–L413) of the cryo-EM structure of HsLRRC8A in 50 mM and 300 mM NaCl, respectively. The initial models were inserted in a 120 Å × 120 Å palmitoyl oleoyl phosphatidyl choline (POPC) bilayer and then solvated in TIP3P waters (box size is 120 Å × 120 Å × 160 Å), with 50 mM or with 300 mM NaCl. To avoid unreasonable starting distributions, all water molecules within the z axis range of the POPC bilayer were removed using an in-house script and ions were introduced into each simulation box by replacing the positions of existing water molecules in a random manner. Therefore, the pore of the constructed systems was free of ions. The initial models of HsLRRC8A in 50 mM and 300 mM NaCl consist of 202,168 and 201,364 atoms, respectively. The initial models of HsLRRC8A mutants were built the same way as the wild type channel.

Three independent simulations were performed for each model using GROMACS 2020 package[54] with isothermal–isobaric (NPT) ensemble and periodic boundary condition. The CHARMM36-CMAP force field[30] was applied to protein, POPC phospholipids, ions and water molecules. For each model, stepwise energy minimizations were performed to relieve unfavorable contacts with positional restraints imposed on the following order: first, protein and lipids, then, protein, then, mainchain atoms of protein, then, Cα atoms of protein, and finally no atoms. Subsequently, three parallel, independent 50-ns equilibration simulations with NPT ensemble were performed for each model with positional restraints applied in the same order as in the energy minimization stage. At this stage, different initial velocities were assigned to atoms in each run by using a random seed to generate velocity according to Maxwell distribution. As a result, ions with distinct initial velocities moved into the pore randomly. This protocol ensured the random initial distribution of ions in the pore, and guaranteed sampling adequacy and reproducibility of our MD simulations. After equilibration, 2.5-μs production run was carried out for each simulation. SETTLE constraints[63] and LINCS constraints[64] were applied on the hydrogen-involved covalent bonds in water molecules and in other molecules, respectively, and the time step was set to 2 fs. Electrostatic interactions were calculated with the Particle-Mesh Ewald (PME) algorithm[65] with a real-space cutoff of 1.0 nm. The temperature was maintained at 310 K using the v-rescale method[66] and the pressure was kept constant at 1 bar by semi-isotropic coupling to a Parrinello-Rahman barostat[67] with $|\tau_p = 2.5$ ps and a compressibility of 4.5×10^{-5} bar. Analysis of simulation data was performed using PyMOL (http://www.pymol.org), GROMACS[54] tools, and in-house scripts.

Steered molecular dynamics simulations

A steered molecular dynamics (SMD) simulation method[68] was employed to sample the trajectory of a single traversing Na^+ or Cl^- ion using GROMACS 2020 package.[54] Each initial model of wild type HsLRRC8A channel or the related mutants were taken from the snapshots that are after 50-ns equilibration simulations with NPT ensemble in 300 mM NaCl. Then, Na^+ or Cl^- was pulled through the channel pore (along the z axis, which is also the symmetry axis of the hexameric channel) using a harmonic force constant of 100 kJ mol^{-1} nm^{-2} and a pulling speed of 0.0025 nm ps^{-1} for 5 ns (total path length is 125.0 Å). The mass center of the pore domain was used as the reference for the pulling SMD to ensure that the ions permeate through the channel's pore domain. The trajectories generated from the SMD simulations provided the reaction coordinates for the potential-of-mean-force (PMF) calculations of single ion permeation.

Potential-of-mean-force calculations

PMFs with respect to Na^+ and Cl^- permeation were calculated using the umbrella sampling method.[69] Windows were generated using snapshots from the pulling SMD trajectories of wild type HsLRRC8A or the channel mutants, with a spacing of approximately 3.0 Å in length (z axis). Na^+ or Cl^- ions were restrained by repulsive boundary potentials in 42 windows along the ~125.0 Å pulling length. The permeating ion was restrained inside the pore near its z axis position using the same force constants as those used for the pulling

SMD. Each window was first equilibrated for 5 ns, then sampled for 20 ns. For each model, it took 1.05 ⌠s for a single ion passing through 42 windows. PMF profiles were then calculated using the Weighted Histogram Analysis Method (WHAM)[70] with bootstrap analysis for statistical errors. All MD umbrella sampling simulations were run using GROMACS 2020[54] package.

Electrophysiology

HCT116 *LRRC8*$^{-/-}$ cells[3] and WT HCT116 cells were maintained in McCoy's 5A medium (PAN Biotech) supplemented with 10% FBS (PAN Biotech) and 1% penicillin/streptomycin (PAN Biotech) at 37°C and 5% CO_2. For electrophysiological recordings, cells were seeded on gelatin-coated coverslips and transfected using Lipofectamine 2000 (Life Technologies) 18–24 h before recording unless stated otherwise. Untagged LRRC8A in pcDNA3.1 (Thermo Fisher Scientific) was co-expressed with GFP-tagged LRRC8C in pEGFP_N1 (Clontech) at a 1:1 ratio, as previously described.[3] GFP fluorescence at the plasma membrane was used to identify doubly transfected cells, as LRRC8C needs co-assembly with LRRC8A for plasma membrane targeting.[3]

VRAC currents were recorded in the standard whole-cell configuration at room temperature using an EPC-10 patch-clamp amplifier and PatchMaster v2x90.3 software (HEKA Elektronik). Signal was sampled at 5 kHz and filtered with a lowpass Bessel filter at 2.9 kHz during acquisition. Voltage clamp protocol contained a 600 ms voltage ramp within a total trace duration of 1 s applied every 10 s. Voltage was held at −30 mV between sweeps.

To measure anion over cation selectivity, we used NaCl-based solutions. Patch pipettes were filled with solution containing 140 mM NaCl, 5 mM EGTA, 3 mM MgATP, and 10 mM HEPES-NMDG (pH 7.2); the isotonic bath solution contained 100 mM NaCl, 112 mM mannitol, 1 mM $MgCl_2$, 1.5 mM $CaCl_2$, 10 mM glucose, and 10 mM HEPES (pH 7.4, 320 mOsm/kg). 25% hypotonic solution contained 100 mM NaCl, 22 mM mannitol, 1 mM $MgCl_2$, 1.5 mM $CaCl_2$, 10 mM glucose, 10 mM HEPES (pH 7.4, 240 mOsm/kg). For measuring shifts in E_{rev}, NaCl concentration was reduced 10-fold and osmolarity was kept constant by adding 180 mM mannitol. This resulted in a reduction in Na^+ concentration from 100 to 10 mM, and Cl^- concentration from 105 to 15 mM. Additionally, a shift in E_{rev} was assessed with a solution having a total Na^+ concentration of 10 mM and Cl^- concentration of 105 mM, which was achieved by partial substitution of NaCl by NMDG-Cl. Liquid junction potentials were calculated according to the stationary Nernst-Planck equation using LJPcalc (https://swharden.com/software/LJPcalc/) and were subtracted during offline analysis. Predicted shifts in reversal potential for purely Na^+-permeable channel would be −59 mV, and 50 mV for a pure Cl^- conductance.

For measuring relative anion permeabilities, patch pipettes were filled with solution containing 40 mM CsCl, 100 mM cesium methanesulfonate, 1 mM $MgCl_2$, 5 mM EGTA, 4 mM Na_2ATP, and 10 mM HEPES (pH 7.2, 290 mOsm/kg) and had a resistance of 2–4 MOhm. The isotonic bath solution contained 150 mM NaCl, 6 mM KCl, 1 mM $MgCl_2$, 1.5 mM $CaCl_2$, 10 mM glucose, and 10 mM HEPES (pH 7.4, 320 mOsm/kg). 25% hypotonic solution contained 105 mM NaCl, 6 mM CsCl, 1 mM $MgCl_2$, 1.5 mM $CaCl_2$, 10 mM glucose, 10 mM HEPES (pH 7.4, 240 mOsm/kg). To study the influence of intracellular ionic strength on permeability ratios cesium methanesulfonate was substituted with 180 mM mannitol.

To obtain shifts in reversal potential, NaCl in the hypotonic solution was substituted with equimolar amount of NaI or NaF. Relative anion permeabilities (P_I/P_{Cl}) were calculated from the shifts of reversal potential using a modified Goldman–Hodgkin–Katz equation $\frac{P_X}{P_{Cl}} = \frac{[Cl]_{hypo}e^{-\Delta E_{rev}F/RT} - [Cl]_{subst}}{[X]_{subst}}$, where ΔE_{rev} is the shift in reversal potential, $[Cl]_{hypo}$ and $[Cl]_{subst}$ are the extracellular Cl^- concentrations in the normal and anion-substituted hypotonic saline, and $[X]_{subst}$ is the concentration of the substituting anion. R is the gas constant, T is the absolute temperature, and F is the Faraday constant. Liquid junction potentials were calculated, but not considered in analysis, as difference in liquid junction potentials between Cl^- and I^--containing solutions is estimated to 0.01 mV.

Half-activation time was estimated from the time course of current activation (estimated at +100 mV from voltage ramps) fitted by a logistic equation $y = \frac{1}{1+e^{-k(x-x_0)}}$, by finding the time at which the equation equals to 0.5.

QUANTIFICATION AND STATISTICAL ANALYSIS

Analysis of electrophysiology experiments was performed using SciPy 1.5.2 library[71] for Python 3.8 programming language (Python Software Foundation). Statistical significance was assessed by the Mann-Whitney U test, false discovery rate was controlled by Benjamini-Hochberg procedure, and p values were corrected accordingly.

Cell Reports, Volume *42*

Supplemental information

Structural insights into anion selectivity and activation mechanism of LRRC8 volume-regulated anion channels

Heng Liu, Maya M. Polovitskaya, Linlin Yang, Meiling Li, Hongyue Li, Zhen Han, Jianguo Wu, Qiansen Zhang, Thomas J. Jentsch, and Jun Liao

Figure S1

```
                   NT                   TM1                  E1β
LRRC8A_Homo      MIPVTELRYFADTQPAYRILKPWWDVFTDYISIVMLMIAVFGGTLQVTQDKMI-CLPCKW  59
LRRC8A_Mus       MIPVTELRYFADTQPAYRILKPWWDVFTDYISIVMLMIAVFGGTLQVTQDKMI-CLPCKW  59
LRRC8A_Gallus    MIPVTELRYFADTQPAYRILKPWWDVFTDYISIVMLMIAVFGGTLQVTQDKMI-CLPCKW  59
LRRC8A_Xenopus   MIPVTELRYFADTQPAYRILKPWWDVFTDYISIVMLMIAVFGGTLQVTQDKMI-CLPCKW  59
LRRC8D_Homo      MFTLAEVASLNDIQPTYRILKPWWDVFMDYLAVVMLMVAIFAGTMQLTKDQVV-CLPVLP  59
LRRC8C_Homo      MIPVTEFRQFSEQQPAFRVLKPWWDVFTDYLSVAMLMIGVFGCTLQVMQDKII-CLPKRV  59
LRRC8E_Homo      MIPVAEFKQFTEQQPAFKVLKPWWDVLAEYLTVAMLMIGVFGCTLQVTQDKII-CLPNHE  59
LRRC8B_Homo      MITLTELKCLADAQSSYHILKPWWDVFWYYITLIMLLVAVLAGALQLTQSRVLCCLPCKV  60

LRRC8A_Homo      VTKDSCNDSFRGWAA-PG--------------------------PEPTYP------N--  83
LRRC8A_Mus       VTKDSCNDSFRGWAA-SN--------------------------PEPTYP------N--  83
LRRC8A_Gallus    ITKDSCNDTVRGWTA-VT--------------------------PERIYY------N--  83
LRRC8A_Xenopus   VTHDSCNDSYRAWNV-PE--------------------------TD-LYT------N--  82
LRRC8D_Homo      SPVNSKAHTPPGNAEVTTNIPKMEAATNQDQDGRTTNDISFGTSAVTPDIPLRATYPRTD  119
LRRC8C_Homo      QPAQNHSSLSNVSQAVAS--------------------------TTPLPPP-----K--  85
LRRC8E_Homo      LQENL-------SEA-PC--------------------------QQLLPRG-----I--  77
LRRC8B_Homo      EFDNHCAVPWDILKA-SM--------------------------NTSSNP---------  83
                                            E1H          TM2
LRRC8A_Homo      ----STILPTPDTGPTGIKYDLDRHQYNYVDAVCYENRLHWFAKYFPYLVLLHTLIFLAC  139
LRRC8A_Mus       ----STVLPTPDTGPTGIKYDLDRHQYNYVDAVCYENRLHWFAKYFPYLVLLHTLIFLAC  139
LRRC8A_Gallus    ----SSLVPSPDTGPTGIQYDLDRHQYNYVDAVCYENRLHWFAKYFPYLVLLHTLIFLAC  139
LRRC8A_Xenopus   ----STLSPPLAPGPTGIKYDLDRHQYNYVDAVCYENRLHWFAKYFPYLVLLHTLIFLAC  138
LRRC8D_Homo      FALPNQEAKKEKKDPTGRKTNLDFQQYVFINQMCYHLALPWYSKYFPYLALIHTIILMVS  179
LRRC8C_Homo      ----PSPANPITVEMKGLKTDLDLQQYSFINQMCYERALHWYAKYFPYLVLIHTLVFMLC  141
LRRC8E_Homo      ----P-EQIGALQEVKGLKNNLDLQQYSFINQLCYETALHWYAKYFPYLVVIHTLIFMVC  132
LRRC8B_Homo      --------GTPLPLPLRIQNDLHRQQYSYIDAVCYEKQLHWFAKFFPYLVLLHTLIFAAC  135
                 TM2      IL1H1
LRRC8A_Homo      SNFWFKFPRTSSKLEHFVSILLKCFDSPWTTRALSETVVEESDPKPAFSKMN-GSMDKKS  198
LRRC8A_Mus       SNFWFKFPRTSSKLEHFVSILLKCFDSPWTTRALSETVVEESDPKPAFSKMN-GSMDKKS  198
LRRC8A_Gallus    SNFWFKFPRTSSKLEHFVSILLKCFDSPWTTRALSETVVEESDTKPAFGKMN-GSMDKKS  198
LRRC8A_Xenopus   SNFWFKFPRTSSKLEHFVSILLKCFDSPWTTRALSETVVEESDPKPTGGKMN-GSVDKKS  197
LRRC8D_Homo      SNFWFKYPKTCSKVEHFVSILGKCFESPWTTKALSETACEDSEENKQRITGAQTLP-KHV  238
LRRC8C_Homo      SNFWFKFPGSSSKIEHFISILGKCFDSPWTTRALSEVSGEDSEEKDNRKNNMNRSNTI-Q  200
LRRC8E_Homo      TSFWFKFPGTSSKIEHFISILGKCFDSPWTTRALSEVSGENQKGPAATERAAATIVAMAG  192
LRRC8B_Homo      SNFWLHYPSTSSRLEHFVAILHKCFDSPWTTRALSETVAEQSVRPLKLSKSK-IL--LSS  192
                                                                IL1H2
LRRC8A_Homo      STVSEDV--EAT--VPMLQRTKSRIEQGIVDRSETGVLDKKEGEQAKALFEKVKKFRTHV  254
LRRC8A_Mus       STVSEDV--EAT--VPMLQRTKSRIEQGIVDRSETGVLDKKEGEQAKALFEKVKKFRTHV  254
LRRC8A_Gallus    STVSEDV--EAT--VPMLQRTKSRIEQGIVDRSETGVLDKKEGEQAKALFEKVKKFRTHV  254
LRRC8A_Xenopus   STASEDV--EAT--VPMLQRSKSRVEQGIVDRSETGVLDKKEGEQAKALFEKVKKFRTHV  253
LRRC8D_Homo      STSSDEGSPSASTPMINKTGFKFSAEKPVIEVPSMTILDKKDGEQAKALFEKVRKFRAHV  298
LRRC8C_Homo      SGP---E--GS---LVNSQSLKSIPEKFVVDKSTAGALDKKEGEQAKALFEKVKKFRLHV  252
LRRC8E_Homo      TGP---G--KAG--EGEKEKVLAEPEKVVTEPPVVTLLDKKEGEQAKALFEKVKKFRMHV  245
LRRC8B_Homo      SGCSADI--DSG--KQSLPYPQPGLESAGIESPTSSVLDKKEGEQAKAIFEKVKRFRMHV  248
                               TM3                  E2β1      E2β2
LRRC8A_Homo      EEGDIVYRLYMRQTIIKVIKFILIICYTVYYVHNIKFDVDCTVDIESLTGYRTYRCAHPL  314
LRRC8A_Mus       EEGDIVYRLYMRQTIIKVIKFVLIICYTVYYVHNIKFDVDCTVDIESLTGYRTYRCAHPL  314
LRRC8A_Gallus    EEGDIVYRLYMRQTIIKVIKFILIICYTVYYVNNITFDVDCKVDIESLTGYRMYRCAHPL  314
LRRC8A_Xenopus   EEGDIVYRLYMRQTIIKVIKFIIILCYTVYYVSSIKFDVDCKVDIESLTGYRMYRCAHPL  313
LRRC8D_Homo      EDSDLIYKLYVVQTVIKTAKFIFILCYTANFVNAISFEHVCKPKVEHLIGYEVFECTHNM  358
LRRC8C_Homo      EEGDILYAMYVRQTVLKVIKFLIIIAYNSALVSKVQFTVDCNVDIQDMTGYKNFSCNHTM  312
LRRC8E_Homo      EEGDILYTMYIRQTVLKVCKFLAILVYNLVYVEKISFLVACRVETSEVTGYASFCCNHTK  305
LRRC8B_Homo      EQKDIIYRVYLKQIIVKVILFVLIITYVPYFLTHITLEIDCSVDVQAFTGYKRYQCVYSL  308
                           TM4                IL2H1
LRRC8A_Homo      ATLFKILASFYISLVIFYGLICMYTLWWMLRRSLKKYSFESIREESSYSDIPDVKNDFAF  374
LRRC8A_Mus       ATLFKILASFYISLVIFYGLICMYTLWWMLRRSLKKYSFESIREESSYSDIPDVKNDFAF  374
LRRC8A_Gallus    ATLFKILASFYISLVVVYGLICMYTLWWMLRRSLKKYSFESIREESSYSDIPDVKNDFAF  374
LRRC8A_Xenopus   ATLFKILASFYISLVGFYGLVCVYTLWWMLRRSLKKYSFESIREESSYSDIPDVKNDFAF  373
LRRC8D_Homo      AYMLKKLLISYISIICVYGFICLYTLFWLFRIPLKEYSFEKVREESSFSDIPDVKNDFAF  418
LRRC8C_Homo      AHLFSKLSFCYLCFVSIYGLTCLYTLYWLFYRSLREYSFEYVRQETGIDDIPDVKNDFAF  372
LRRC8E_Homo      AHLFSKLAFCYISFVCIYGLTCIYTLYWLFHRPLKEYSFRSVREETGMGDIPDVKNDFAF  365
LRRC8B_Homo      AEIFKVLASFYVILVILYGLTSSYSLWWMLRSSLKQYSFEALREKSNYSDIPDVKNDFAF  368
                 IL2H2    IL2H3         IL2H4
LRRC8A_Homo      MLHLIDQYDPLYSKRFAVFLSEVSENKLRQLNLNNEWTLDKLRQRLTKNAQDKLELHLFM  434
LRRC8A_Mus       MLHLIDQYDPLYSKRFAVFLSEVSENKLRQLNLNNEWTLDKLRQRLTKNAQDKLELHLFM  434
LRRC8A_Gallus    MLHLIDQYDPLYSKRFAVFLSEVSENKLRQLNLNNEWTLEKLRQRITKNSQDKLELHLFM  434
LRRC8A_Xenopus   MLHLIDQYDPLYSKRFAVFLSEVSENKLRQLNLNHEWTLDKLRQRITKNSQEKLELHLFM  433
LRRC8D_Homo      LLHMVDQYDQLYSKRFGVFLSEVSENKLREISLNHEWTFEKLRQHISRNAQDKQELHLFM  478
LRRC8C_Homo      MLHMIDQYDPLYSKRFAVFLSEVSENKLKQLNLNNEWTPDKLRQKLQTNAHNRLELPLIM  432
LRRC8E_Homo      MLHLIDQYDSLYSKRFAVFLSEVSESRLKQLNLNHEWTPEKLRQKLQRNAAGRLELALCM  425
LRRC8B_Homo      ILHLADQYDPLYSKRFSIFLSEVSENKLKQINLNNEWTVEKLKSKLVKNAQDKIELHLFM  428
LRRC8A_Danio     MLHMIDQYDPLYSKRFAVFLSEVSENKLRQLNLNNEWTLEKLRQRITKNSQEKLELHLFM  420
```

Figure S1. Amino-acid sequence alignment of pore domains of LRRC8A orthologs and human LRRC8 paralogs, related to Figures 1–3.

The residues M1–M434 of the pore domain of HsLRRC8A are aligned with the equivalent residues of other orthologs and human LRRC8 paralogs. Residues of NT and TMs that are involved in polar interactions are highlighted in yellow. Secondary structures are marked for HsLRRC8A.

Figure S2

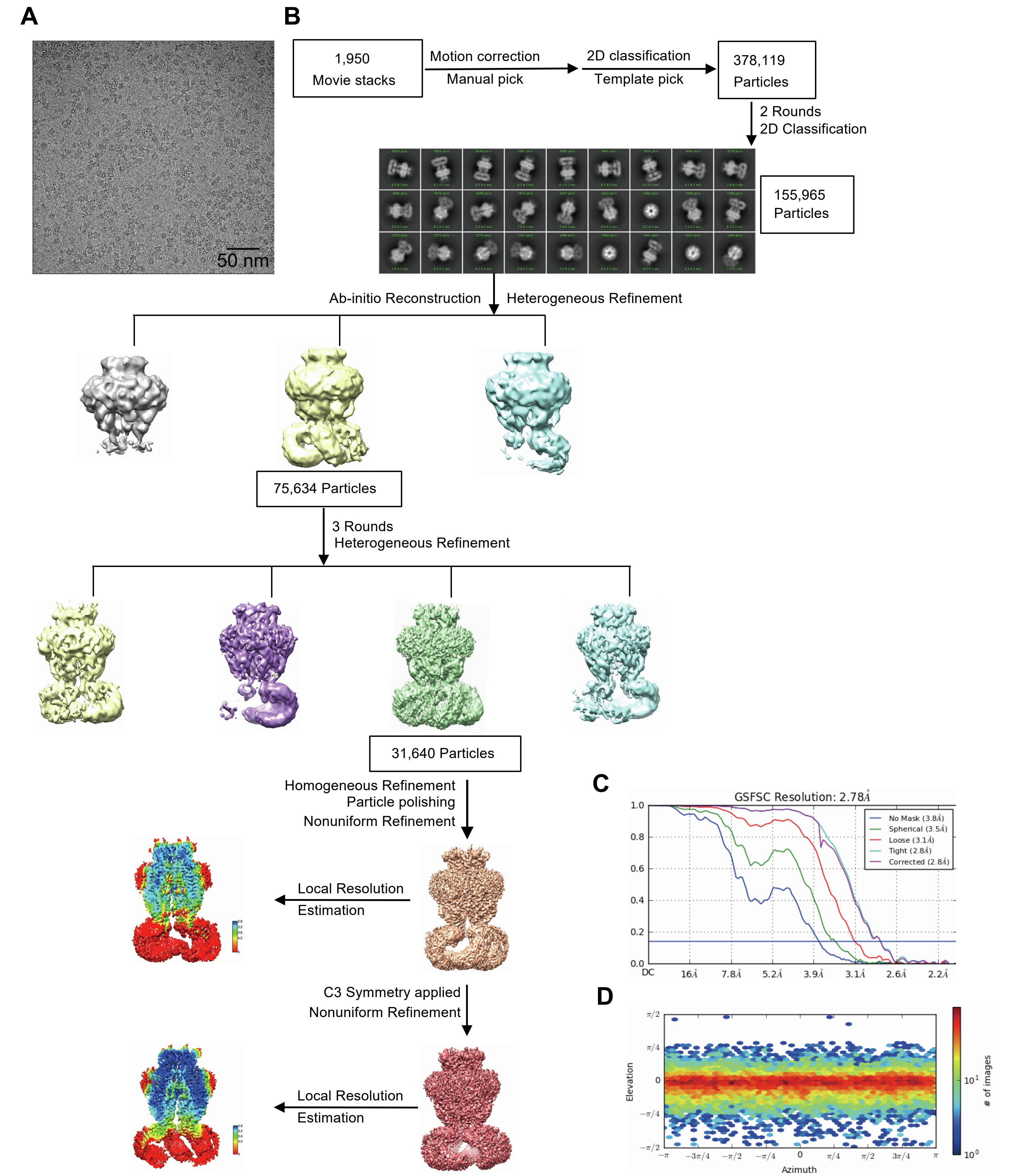

Figure S2. Reconstruction of cryo-EM structure of HsLRRC8A, related to Figures 1 and 2, and Table S1.

(A) Representative cryo-EM micrograph of dataset obtained at FEI Titan Krios. (B) Flowchart of HsLRRC8A reconstruction. Local resolution estimation is shown at the bottom panel. Flowchart of cryo-EM data processing of the HsLRRC8A structure, including particle picking, classification, and 3D refinement. (C) Fourier shell correlation (FSC) of the final 3D reconstruction following gold standard refinement. FSC curves are plotted before and after masking. (D) Angular distribution heatmap of particles used for the refinement.

Figure S3

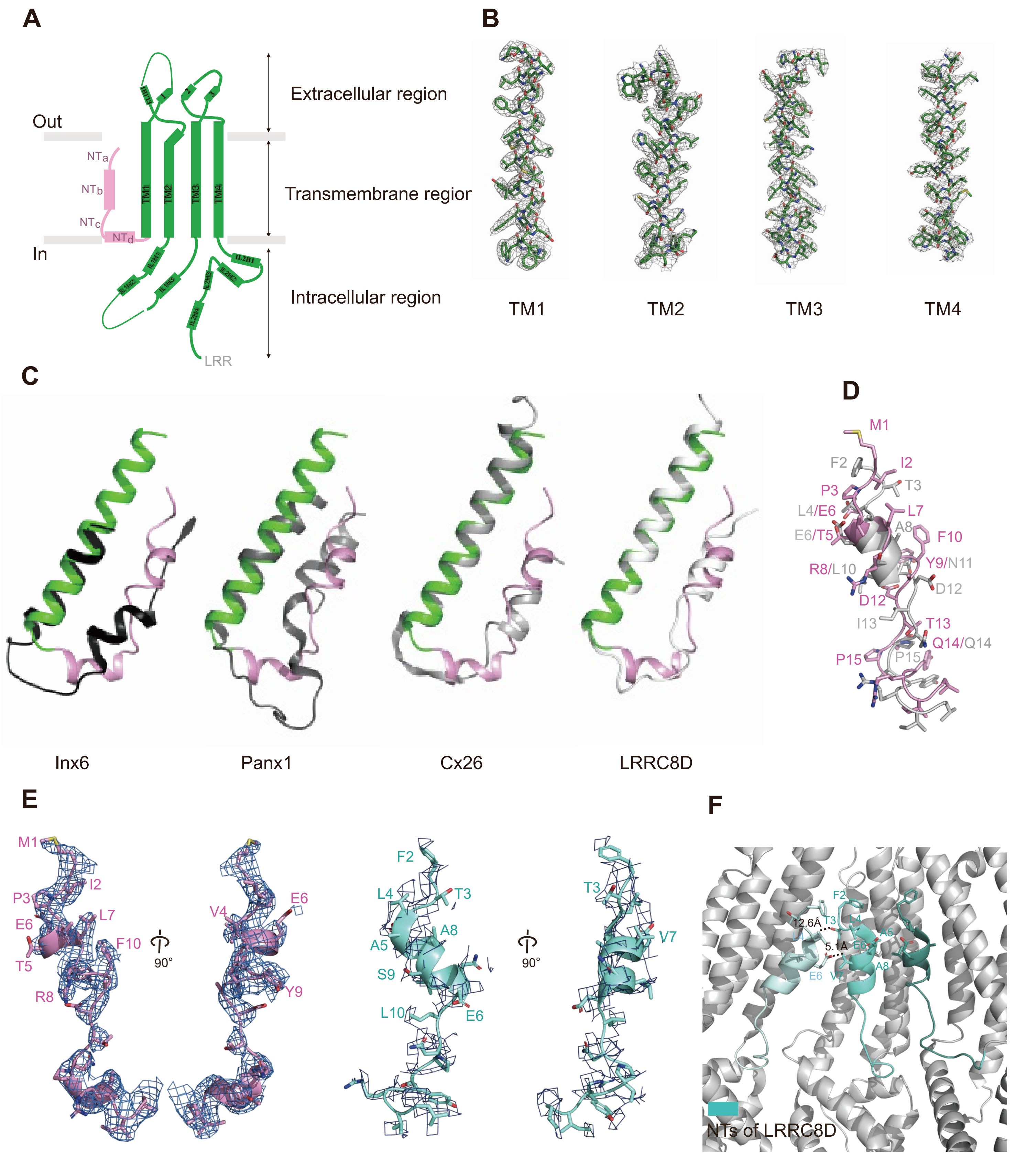

Figure S3. Subunit structure of HsLRRC8A, related to Figures 1 and 2.

(A) Schematic representation of the HsLRRC8A topology for the pore domain. (B) Cryo-EM density map (gray) for TMs. (C) Superimposition between HsLRRC8A NT and those of other large pore channels. PDB codes used in superimposition: INX6, 5H1Q[34]; Pannexin1, 6WBF[35]; Cx26, 2ZW3[33]; LRRC8D, 6M04[15]. (D) Superimposition of the HsLRRC8A NT (colored pink) and HsLRRC8D NT (colored grey), using the Cα atoms of the protomers. (E) Cryo-EM densities (contour level of each map is 2.0 σ) of NTs from a HsLRRC8A protomer (left) and a HsLRRC8D protomer (right), viewed from same orientations. (F) HsLRRC8D NTs and their local surroundings. No evident nonpolar and polar interactions were found between adjacent NTs.

Figure S4.

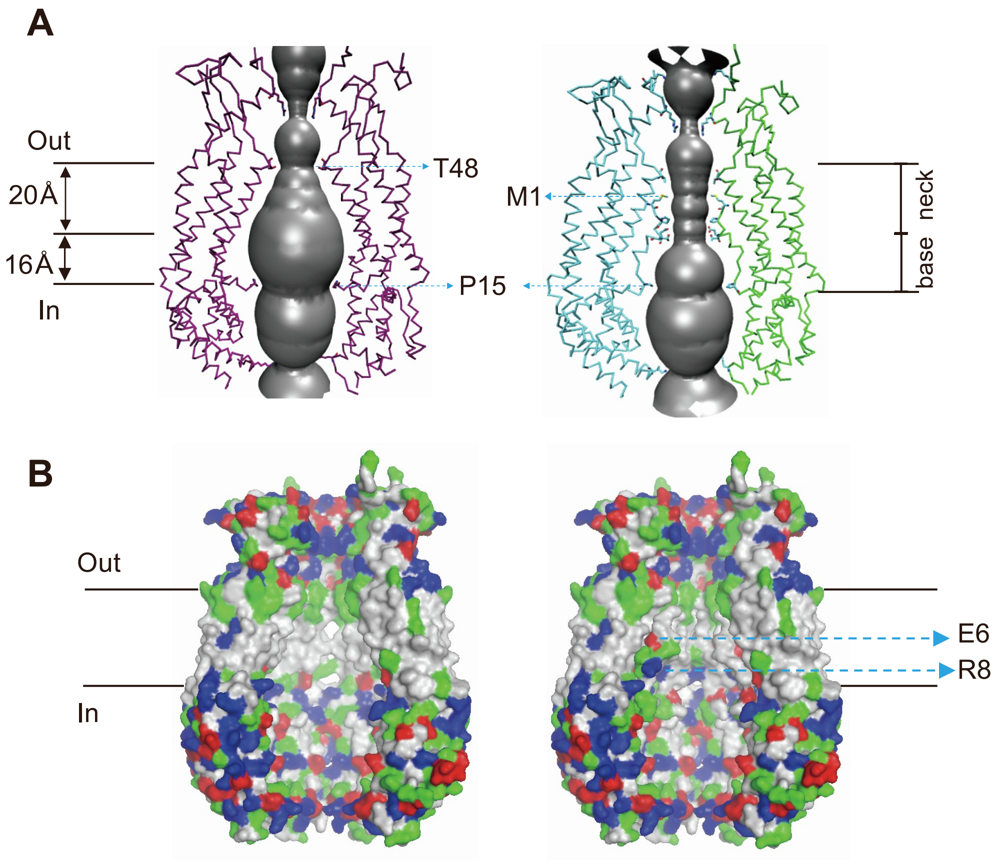

Figure S4. Permeation paths of LRRC8A channels containing unresolved NTs (left) and resolved NTs (right), related to Figures 1 and 2.

(A) Permeation paths relative to two opposite subunits. The neck and base have been marked. (B) Molecular surface of the permeation path viewed from the membrane. The surface is colored according to chemical properties of residues (hydrophobic, gray; hydrophilic, green; acidic, red; basic, blue). The PDB code is 5ZSU for HsLRRC8A containing the unsolved NTs. The two front subunits are removed for clarity.

Figure S5

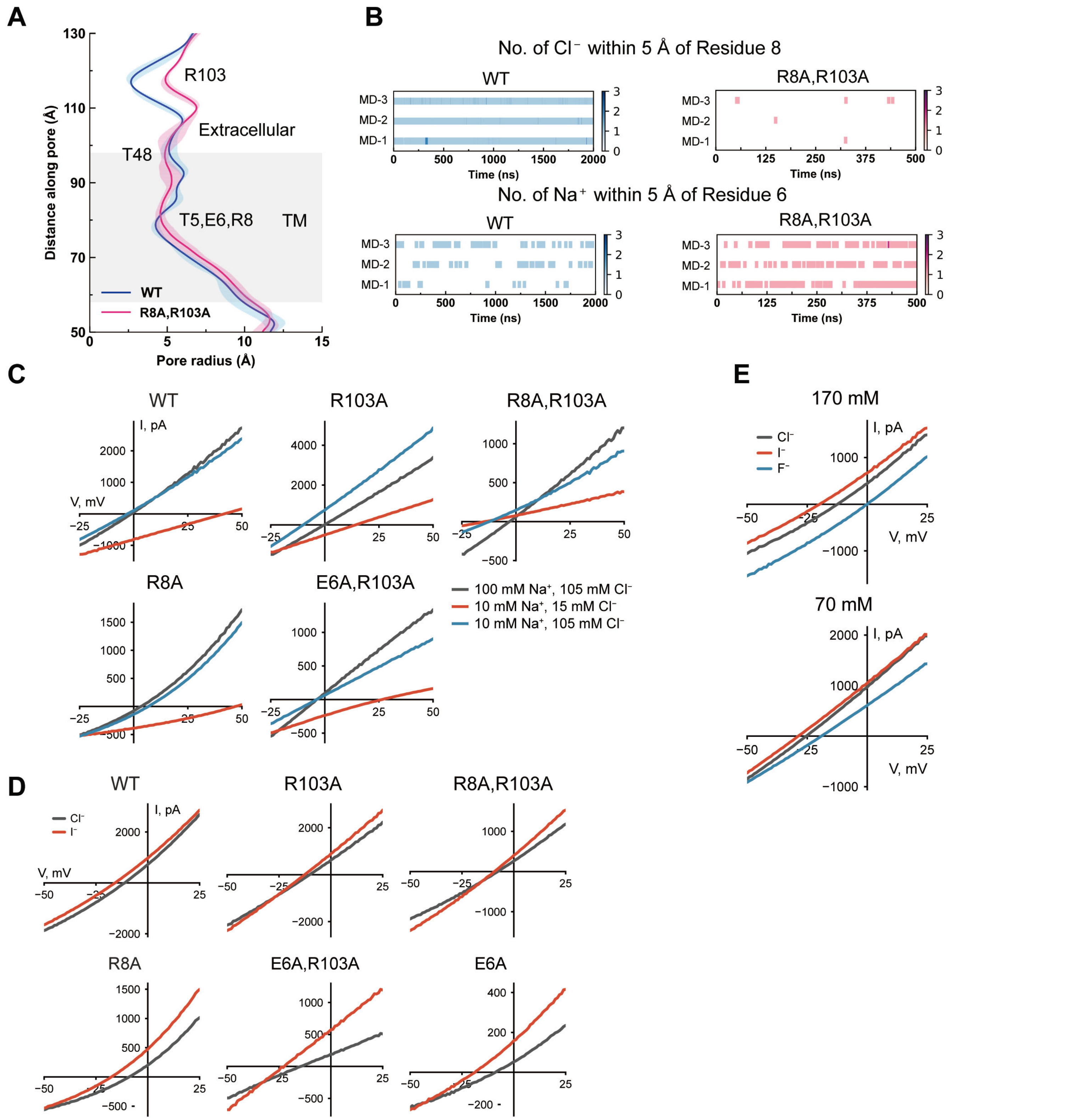

Figure S5. Influence of mutations on pore properties of LRRC8A channel as predicted by MD simulations (A and B) and tested by electrophysiology (C-E), related to Figure 3.
(A) Pore radius along the symmetry axis in WT (blue) and mutant (magenta) channels, respectively. The transmembrane segment of the pore domain is colored in grey. Radii were calculated from snapshots at 1-ns intervals in the last 1000-ns simulations. Data are shown as mean ± s.d. of three independent simulations for each system. (B) Number of Cl^- near residue 8 (top) and number of Na^+ near residue 6 (bottom) in WT (left) and the denoted mutant (right) channels during the course of simulations. (C) Averaged current traces from the recordings shown in Figures 3I and 3J, demonstrating the shifts in reversal potential between the high NaCl bath solution (gray), the low NaCl bath solution (red), and the low Na^+ (blue) bath solution. (D) Averaged current traces from the recordings shown in Figures 3K, 3L, and 3M, demonstrating the shifts in reversal potential between the NaCl (gray) and NaI (red) bath solutions. (E) Averaged current traces from the recordings shown in 4M and 4N, demonstrating the shifts in reversal potential between the NaCl- (gray), NaI- (red), and NaF-containing (blue) bath solutions.

Figure S6

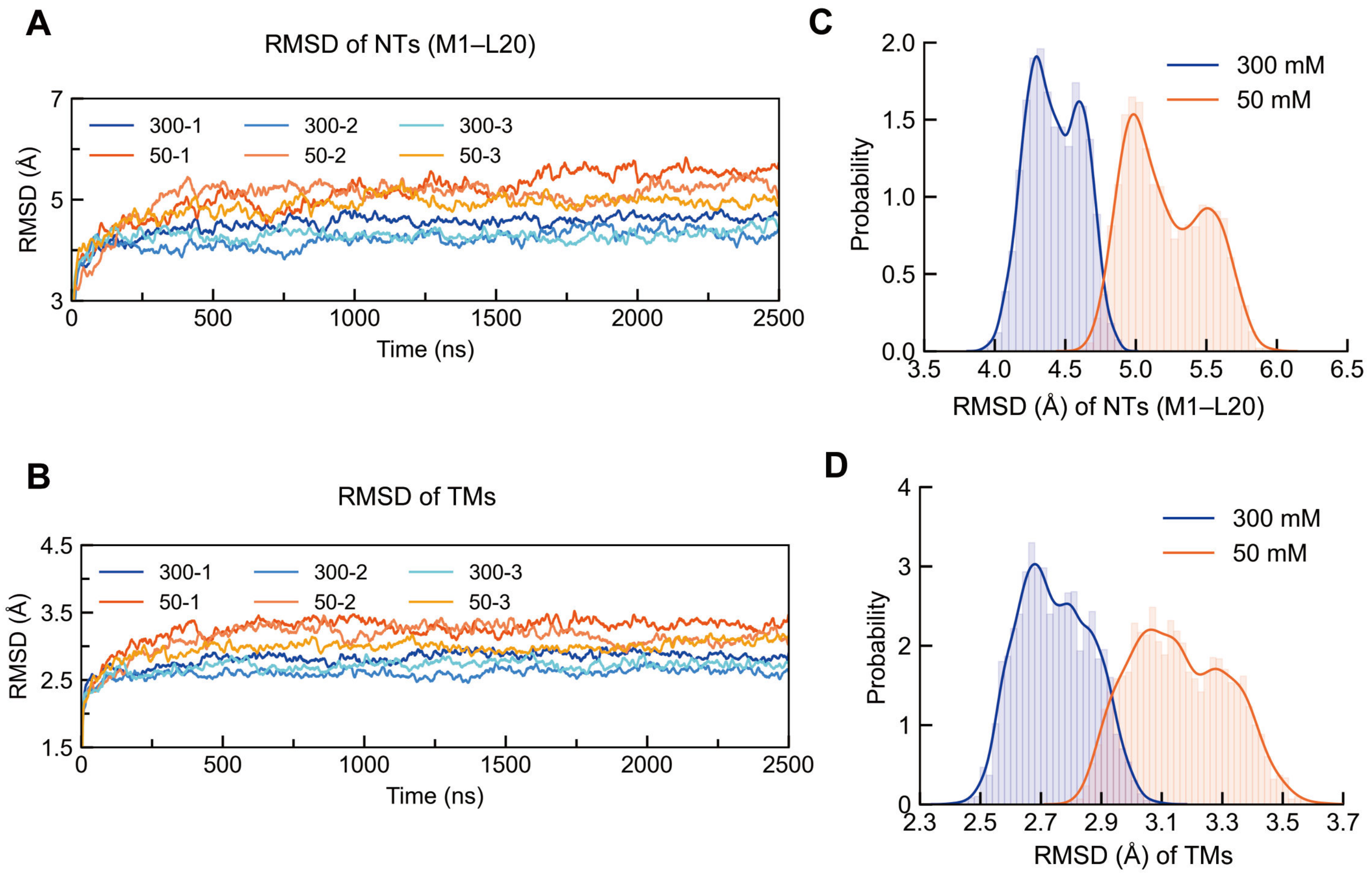

Figure S6. 2500-ns MD trajectories of RMSD values for residues of pore domain and their probability distributions generated from the last 1000-ns simulations, related to Figure 4.

The residue ranges of TMs were defined as: TM1, W23–T48; TM2, W120–K145; TM3, I259–N288; TM4, L314–M343. RMSD values of residues were calculated from their mainchain atoms sampled from snapshots at 100-ps intervals. (A and B) Trajectories of RMSD values for residues of NTs (A) or of TMs (B) in 2500-ns trajectories simulated at salt concentrations of 300 mM and 50 mM NaCl, respectively. Three independent simulations were performed at each salt concentration. (C and D) Probability distributions of RMSD values for residues of NTs (C) or of TMs (D) generated from the last 1000-ns trajectories.

Figure S7

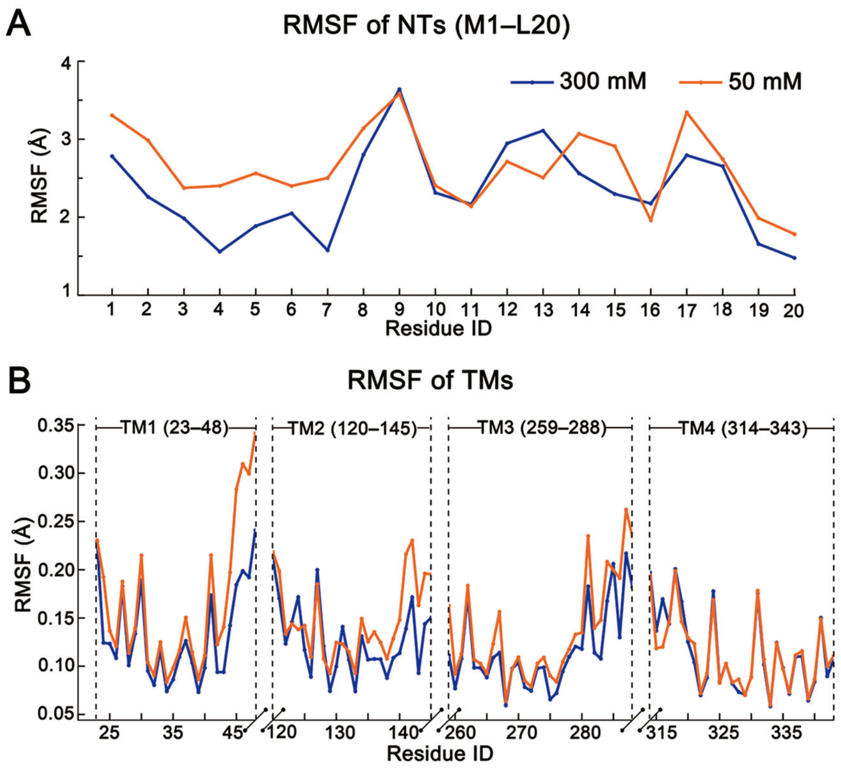

Figure S7. Root mean square fluctuations (RMSF) of each residue of NTs and TMs at 300 mM and 50 mM NaCl, related to Figure 4.

Root mean square fluctuations (RMSF) of each residue of NTs (A) and TMs (B) at 300 mM and 50 mM NaCl. Each subunit in a hexameric HsLRRC8A channel was treated equally. The RMSF value of each residue was calculated based on 18,000 sampled snapshots from all three parallel simulations, as the last 1000-ns snapshots at 1-ns intervals for each of the six subunits were used.

Figure S8

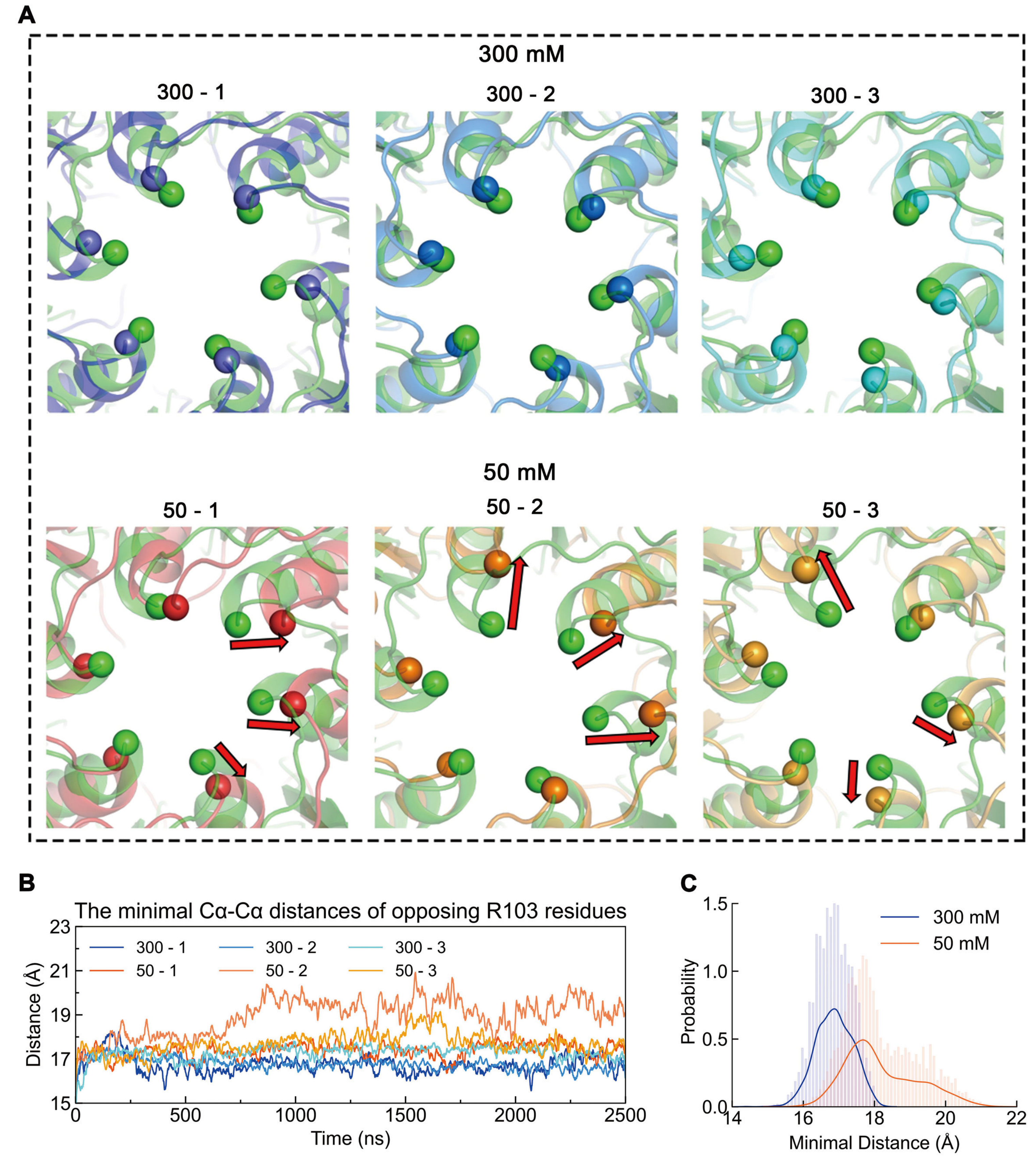

Figure S8. Radial movements of R103 in MD simulations conducted at 300 mM and 50 mM NaCl concentrations, related to Figure 4.

(A) Superposition of representative structures obtained at each salt concentration with the cryo-EM structure (colored in green) of HsLRRC8A, viewed from the top. Cα atoms of R103 residues were depicted as spheres. The red arrows indicate the radial dilation of Cα atoms of R103 residues at 50 mM NaCl. (B) The time evolution of minimal Cα-Cα distances between opposing R103 residues. (C) The probabilities of the Cα-Cα minimal distances calculated for the last 1000-ns simulations.

Figure S9

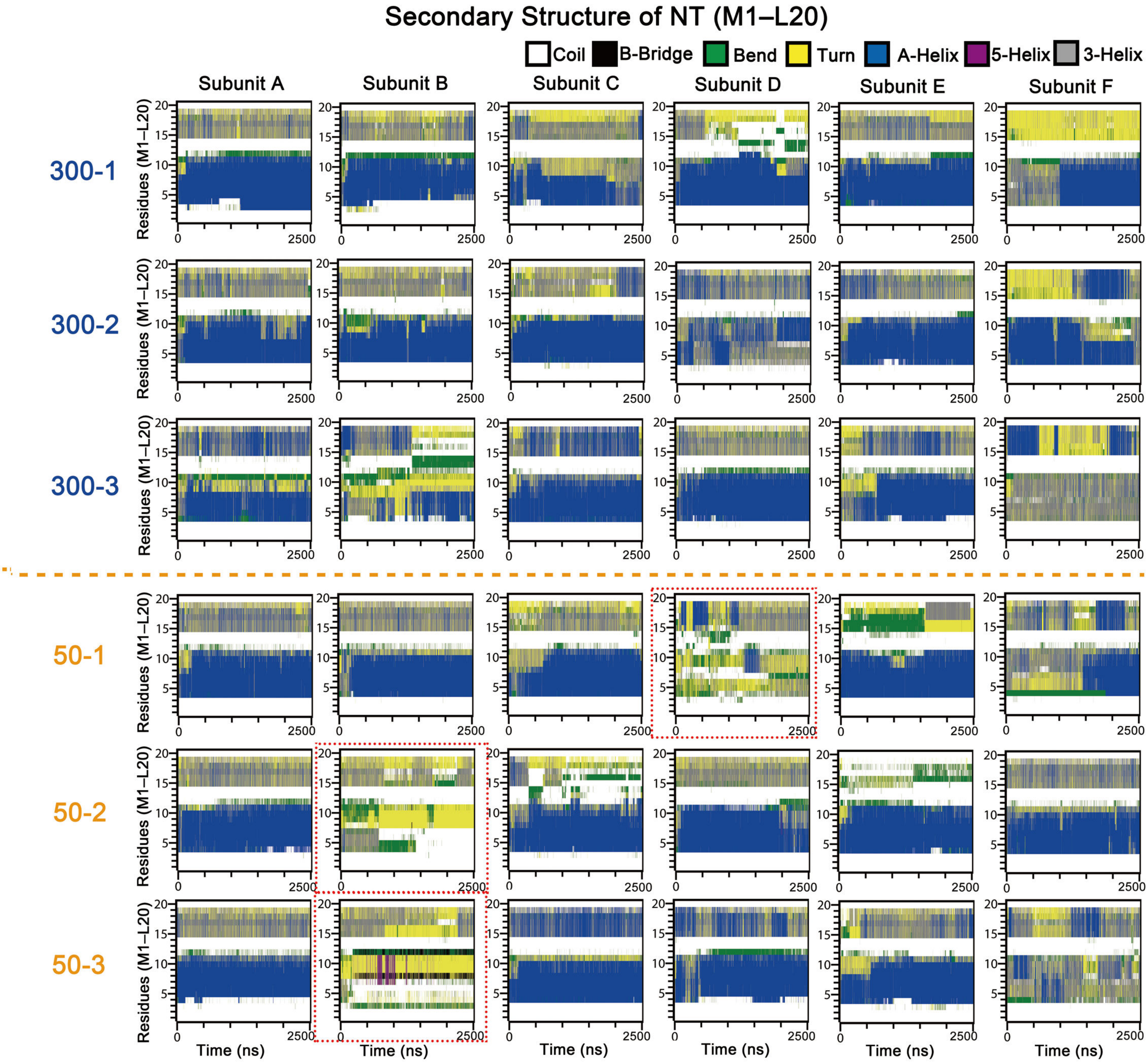

Figure S9. The changes in secondary structures of NT subsegments during the course of simulations, related to Figure 4.

The unwound subsegments at 50 mM NaCl are highlighted by red dashed boxes.

Table S1. Cryo-EM data collection, refinement, and validation statistics, related to Figure 1 and Figure S2.

Data collection and processing	
Microscope	FEI Titan Krios
Camera	K3
Voltage (kV)	300kV
Magnification	105K
Pixel size (Å)	0.52 (1.04)
Electron exposure (e–/Å^2)	~60
Defocus range (μm)	–1.0 to –2.2
Symmetry imposed	C3
Initial particle images (no.)	378,119
Final particle images (no.)	31,640
Map resolution (Å)	2.78
FSC threshold	0.143
**Refinement**	
Initial model used (PDB code)	6G9O
d model	3.2
dFSC model (0/0.143/0.5)	2.7/2.8/3.2
Map sharpening B factor (Å^2)	–78
Model composition	
Non-hydrogen atoms	36203
Protein residues	4385
B factor (Å^2)	304.66
R.m.s. deviations	
Bond lengths (Å)	0.009
Bond angles (°)	1.267
Validation	
MolProbity score	1.92
Clash Score	10.34
Poor rotamers (%)	0
Ramachandran plot	
Favored (%)	95.37
Allowed (%)	4.47
Disallowed (%)	0.16

Table S2. Cα-Cα distance between pairs of opposite residues that constitute the constriction sites along the permeation path, related to Figures 1 and 2.

Distance (Å) between Cα atoms of opposite residues	R103	T48	M1 / P3	E6	P15	K235
**HsLRRC8A**	14.7	16.8	22.7 / 17.1	19.9	28.5	24.5
**6G9O**	15.7	16.9	NA	NA	27.5	28.2
**6NZW**	15.5	18.3	NA	NA	28.7	27.7
**6NZZ**	16.1	18.3	NA	NA	32.4	31.9
**5ZSU**	16.2	18.4	NA	NA	NA	33.9
**6DJB**	15.3	18.8	NA	NA	35.5	31.0

Table S3. Buried surface area in the interface between two adjacent subunits of the pore domain, related to Figure 2. The extracellular, transmembrane and intracellular portions of pore domain are same as those in Figure 1.

**Buried surface (Å²)**	**HsLRRC8A**	**6G9O**	**5ZSU (loose/tight interface)**	**6DJB**	**6NZW**	**6NZZ**
**Total**	2180	1683	1701/1051	1668	1590	1537
**Extracellular segment**	920	915	850/805	860	887	920
**Transmembrane segment**	810	380	376/234	302	379	314
**Contribution of N-halves to transmembrane segment**	770	NA	NA	NA	NA	NA
**Contribution of C-halves to transmenbrane segment**	445	NA	NA	NA	NA	NA
**Intracellular segment**	490	328	464/NA	398	311	266
